# The *Vibrio cholerae* Colonization Factor GbpA Possesses a Modular Structure that Governs Binding to Different Host Surfaces

**DOI:** 10.1371/journal.ppat.1002373

**Published:** 2012-01-12

**Authors:** Edmond Wong, Gustav Vaaje-Kolstad, Avishek Ghosh, Ramon Hurtado-Guerrero, Peter V. Konarev, Adel F. M. Ibrahim, Dmitri I. Svergun, Vincent G. H. Eijsink, Nabendu S. Chatterjee, Daan M. F. van Aalten

**Affiliations:** 1 Division of Molecular Microbiology, College of Life Sciences, University of Dundee, Dundee, United Kingdom; 2 Department of Chemistry, Biotechnology and Food Science, Norwegian University of Life Sciences, Ås, Norway; 3 Division of Biochemistry, National Institute of Cholera and Enteric Diseases, Scheme XM, Beliaghata, Kolkata, India; 4 European Molecular Biology Laboratory (EMBL), Outstation Hamburg at DESY, Hamburg, Germany; University of California San Diego, United States of America

## Abstract

*Vibrio cholerae* is a bacterial pathogen that colonizes the chitinous exoskeleton of zooplankton as well as the human gastrointestinal tract. Colonization of these different niches involves an *N*-acetylglucosamine binding protein (GbpA) that has been reported to mediate bacterial attachment to both marine chitin and mammalian intestinal mucin through an unknown molecular mechanism. We report structural studies that reveal that GbpA possesses an unusual, elongated, four-domain structure, with domains 1 and 4 showing structural homology to chitin binding domains. A glycan screen revealed that GbpA binds to GlcNAc oligosaccharides. Structure-guided GbpA truncation mutants show that domains 1 and 4 of GbpA interact with chitin *in vitro*, whereas *in vivo* complementation studies reveal that domain 1 is also crucial for mucin binding and intestinal colonization. Bacterial binding studies show that domains 2 and 3 bind to the *V. cholerae* surface. Finally, mouse virulence assays show that only the first three domains of GbpA are required for colonization. These results explain how GbpA provides structural/functional modular interactions between *V. cholerae*, intestinal epithelium and chitinous exoskeletons.

## Author Summary

*Vibrio cholerae* is the bacterium that causes cholera, a disease endemic in developing countries with poor sanitation. The bacterium colonizes aquatic organisms that serve as a reservoir of transmission to humans. Our work has focused on GbpA, a protein that is secreted by *V. cholerae* and appears to facilitate growth of the bacteria both in the human intestine and on the exoskeletons of marine organisms. We show that the protein possesses an unusual three-dimensional structure consisting of four separate domains. Two of the domains are similar to proteins that are known to bind chitin, an exoskeleton biopolymer, and our data show that these domains indeed harbour the chitin binding properties of GbpA. One of these domains is also capable of binding intestinal mucus. The two remaining domains are required for interacting with the bacterium itself, creating a stable interface between the bacterium and the human/marine host, facilitating colonization. Finally, work with a cholera mouse model shows that only the first three domains of GbpA are required for colonization. These results show how GbpA provides structural/functional modular interactions between *V. cholerae*, the intestinal epithelium and chitinous exoskeletons.

## Introduction

*Vibrio cholerae* is a Gram-negative bacterial pathogen that causes excessive watery diarrhea in humans [1], [2]. The number of reported cases of cholera worldwide averages over 100000 per annum for the last 10 years [1], [3], and is presumed to be exceeded by the number of unreported cases [3], [4]. Most of these cases occur in countries with poor sanitation [5], [6]. *V. cholerae* strains are classified into more than 200 serogroups, with only the serogroups O1 and O139 possessing epidemic potential. It has been shown that the survival of *Vibrio cholerae* in the intestine is dependent on its ability to adhere to and colonize cell surfaces [7], [8]. In aquatic environments, attachment to fish, crustacea and algae enables the bacteria to obtain nutrients, thereby provide a competitive advantage compared to other free-swimming bacteria [8], [9], [10]. Adherence to aquatic organisms is believed to involve a different set of genes and recognition molecules as compared to those used for intestinal colonization. For example, plankton surface colonization by the bacteria is more dependent on the mannose sensitive hemagglutinin (MSHA) [11], [12].

Recent studies have suggested that *Vibrio cholerae* secretes a protein that mediates adhesion in aquatic environments, e.g. to plankton, as well as adhesion to human intestinal cells [7], [10]. This protein, GlcNAc binding protein A (GbpA) binds to *N*-acetylglucosamine (GlcNAc)- containing carbohydrates, such as chitin, and is secreted by the type 2 secretion system [7]. In addition to chitin, GbpA has been shown to bind to mucins [13] that also contain GlcNAc as part of their densely packed network of O-linked glycans (reviewed in [14], [15]). The importance of GbpA for bacterial colonization has been demonstrated for the O395 [7] and N16961 *Vibrio cholerae* strains [16] (representing classical [17] and El Tor [18] biotypes, respectively).

Here, we have studied the molecular basis of the function of GbpA as a colonization factor, using a combination of approaches. Firstly, we determined the three-dimensional structure of GbpA using a combination of X-ray crystallography and small angle X-ray scattering, revealing an unusual, elongated four-domain fold. Two domains appear to be structurally similar to known chitin binding modules, and we show these domains to be responsible for the ability of GbpA to bind chitin. The other two domains possess distant structural homology to bacterial pili binding proteins and serve to bind the *V. cholerae* surface. Finally, complementation studies with truncated forms of GbpA identify the domains responsible for mucin binding and virulence in a *V. cholerae* mouse infection model.

## Results

### GbpA is a multi-domain protein

Full-length GbpA and a range of truncated forms of GbpA (see Supplemental Table S1 in Text S1 for a summary of all constructs used in this study) were cloned and expressed in *E. coli*. The mature form of the protein (residues 24 to 485, GbpA_fl_) was extracted from the periplasm and purified by chromatography. Two C-terminally truncated forms of mature GbpA, comprising amino acids 24–203 (GbpA_D1_), and 24–414 (GbpA_D1–3_), were also produced. Two N-terminally truncated forms of GbpA comprising amino acids 210–414 (GbpA_D2–3_), and 423–485 (GbpA_D4_) were expressed as GST fusion proteins and purified to homogeneity.

**Figure 1:**
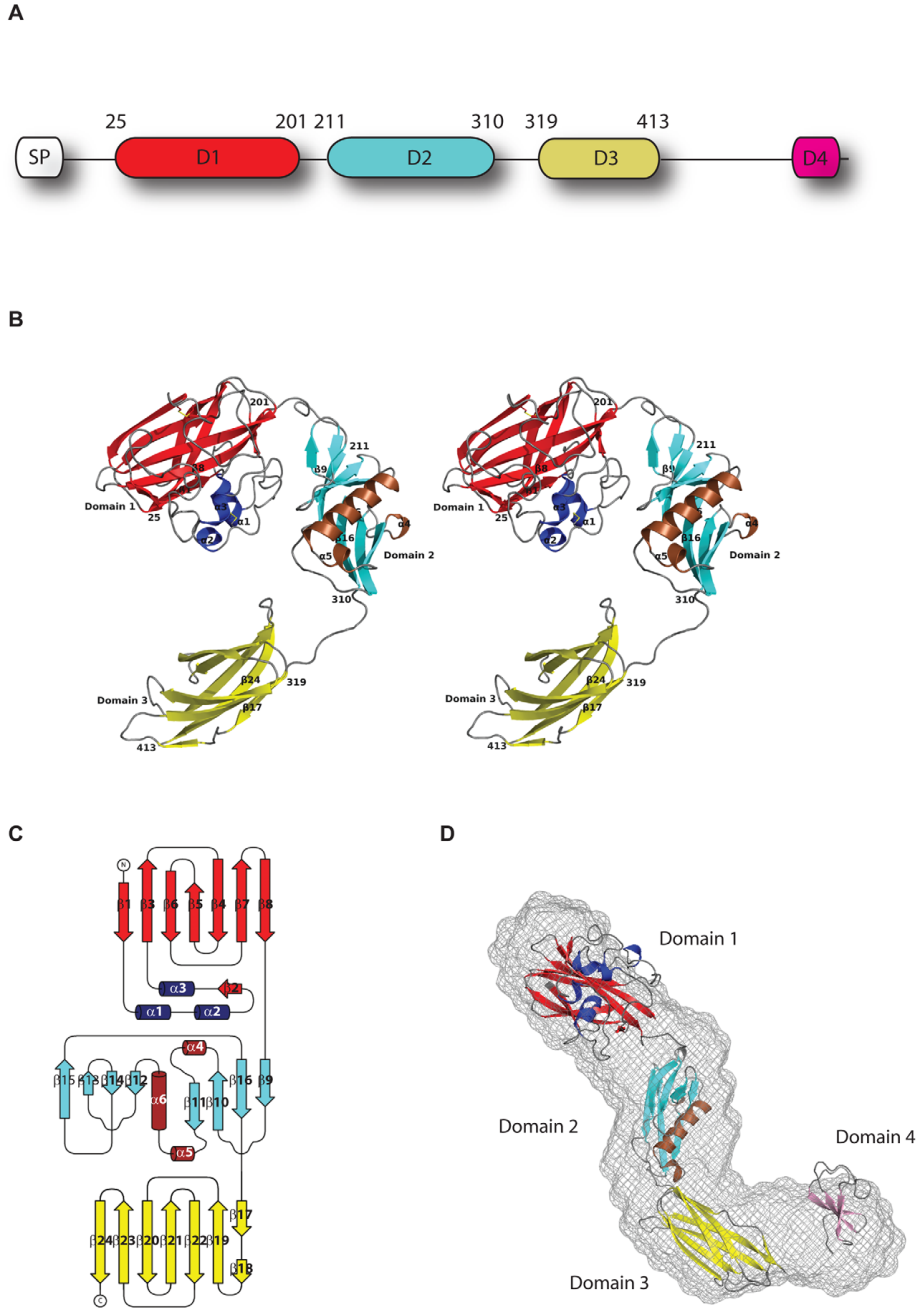
Structure of GbpA. **A.** Schematic representation of the functional domains of GbpA. The residue boundaries for each domain of the determined structure of GbpA are numerically labelled above the diagram. The N-terminus SP box refers to the signal peptide that signals the protein for secretion through the type-2 secretory pathway. **B.** Stereo images of the crystallised structure of GbpA_D1–3_. The structure is coloured according to the secondary structure for each domain. For domain 1, the α-helices are coloured blue, β-sheets are coloured in red. For domain 2, the α-helices are coloured in brown, and the β-sheets are coloured in cyan. For domain 3, the β-sheets are coloured in yellow. All strands are coloured in grey. The residues defining domain boundaries are labelled accordingly. The disulfide bonds are coloured as two yellow coloured sticks. The first and last β-strands for each domain, and all α-helices are labelled. **C.** Topology diagram of GbpA_D1–3_ drawn using TOPDRAW [52]. The β-stands and the α-helices are numbered sequentially. **D.** The *ab initio* SAXS model of GbpA_fl_ (gray spheres) is superimposed onto the structure of GbpA_D1–3_ and the modelled structure of GbpA_D4_ (ribbon models) as determined by rigid body refinement using SASREF.

Although extensive efforts were made to crystallise full-length GbpA, GbpA_D1–3_ was the longest construct that could be crystallised. The GbpA_D1–3_ structure was solved by SAD phasing, and refined against 1.8 Å synchrotron diffraction data (Supplemental Table S2 in Text S1). The structure of GbpA_D1–3_ reveals three distinct domains rich in β-structure, linked by disordered loops (Figure 1A,B). These three domains, and their similarities to previously reported protein structures, are discussed separately.

### Domain 1 is structurally similar to a CBM33 chitin binding protein

**Figure 2:**
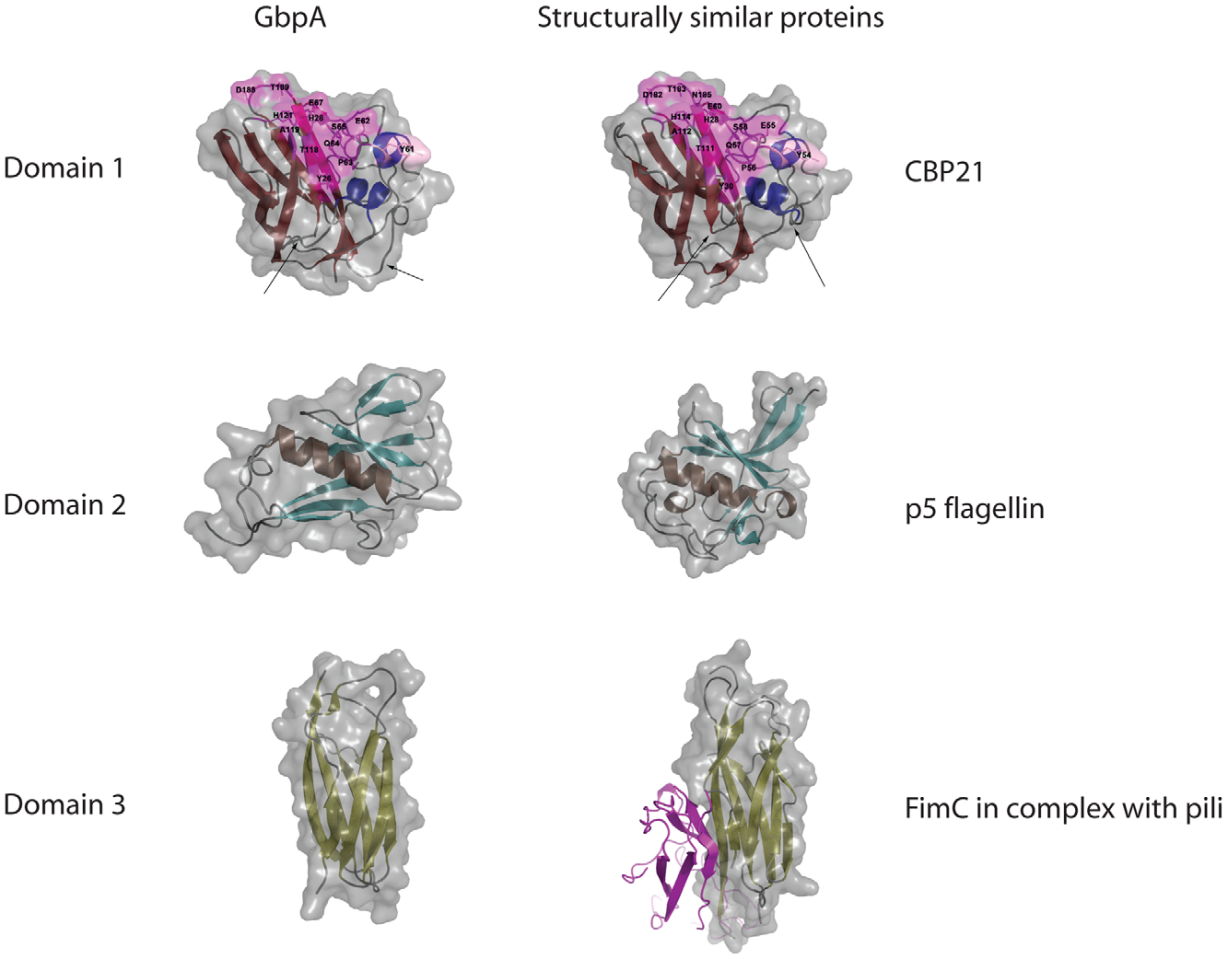
Comparisons of domains 1–3 of GbpA with structural homologues. The space filled models for each domain of the crystal structure of GbpA_D1–3_ are shown superimposed onto the corresponding ribbon models. For each ribbon models, the secondary structure was coloured in the same scheme as Figure 1B. Each domain of GbpA_D1–3_ was parse through a structural alignment server (DALI, [19]). The protein domains giving the best alignment to each domain of GbpA are presented on the right as space filled models superimposed onto their corresponding ribbon models. The individual domains of GbpA are also presented in the same orientation as the structural homologues. For the first row CBP21 (2BEM.pdb) aligned the best to Domain 1 of GbpA. The magenta-coloured surfaces of domain 1 and CBP21 indicate areas of high sequence conservation, with side chains specifically discussed in the text shown as sticks and labelled. The position of CBP21 Y54 (and the GbpA equivalent Y61) that is critical for chitin binding is coloured in pink. Arrows indicate the position of the extended loops in GbpA that are absent in CBP21. For the second row, the flagellin protein p5 (2ZBI.pdb) showed the closest structural alignment to domain 2 of GbpA. For the third row, the pili-binding chaperone FimC (1QUN.pdb) was shown to be structurally similar to domain 3 of GbpA. The structure of FimC was solved in complex with part of the Type 1 pili FimH. In the figure, FimH is shown in magenta.

Domain 1 (GbpA_D1_) comprises a four-stranded and a three-stranded β-sheet forming a β-sandwich (Figure 1B,C). Between β-strands 1 and 3, a 65-residue loop forms a pseudo-domain, which consists of short α-helices, a β-strand, and loops. There are also two disulphide bonds formed: one in the loop/helical region (Cys-42 and Cys-56) and one linking β-strands 4 and 5 (Cys-152 and Cys-169) (Figure 1B). A structural similarity search with the Dali server [19] identified chitin binding protein 21 (CBP21) from *S. marcescens*, which is part of the CAZy Carbohydrate Binding Module family 33 (CBM33 [20]) as a structural homologue (Figure 2, RMSD = 0.9 Å for 168 Cαatoms, 47% sequence identity). Previous work has shown that CBP21 facilitates chitin degradation in the presence of chitinases [21]. Previous work [21], [22] has shown that residues Y54, E55, E60, H114, D182 and N185 are important for chitin binding by CBP21. Interestingly, most of these are conserved in GbpA domain 1 (Figure 2), except N185, equivalent to GbpA A191. Y54 in CBP21 has been shown to play a role in determining binding specificity as well as affinity for different types of chitin [22]. Residue 191 of GbpA is an alanine, and mutation of the equivalent N185 in CBP21 to alanine resulted in a three-fold reduction in chitin affinity [22]. A noticeable difference between CBP21 and GbpA_D1_ are the lengths of two loops: one located between β-strand 1 and α-helices 1 (amino acids 29 to 39), and another between α-helices 1 and α-helices 2 (amino acids 44 to 56) of GbpA (Figure 1B Figure 1C). These loops form a continuous patch on the opposite side from the proposed chitin binding face of domain 1 (Figure 2).

### GbpA domains 2 and 3 possess distant structural similarity to bacterial surface proteins

Structural similarity searches with DALI revealed only distant structural similarities between domain 2 of GbpA and the β-domain of the flagellin protein p5 (Z = 4.0, RMSD = 2.9 Å for 68 Cα atoms, Figure 2). According to Maruyama *et al.*, this domain interacts with the bacterial surface, and functions to project an alginate binding domain of the protein from the cell surface [23]. A more significant structural match was observed for domain 3 of GbpA. This domain has an immunoglobulin fold (Figure 2) with significant structural similarity to over 200 structures in the PDB. The best hit was SFAE (Z = 9.8, RMSD = 2.4 Å for 91 Cα atoms), a chaperone that functions to fold and transport components of *Escherichia coli* surface pili to the cell surface [24]. Structures similar to SFAE have been solved in complex with pili subunits; for instance, the protein FimC (the 4th best DALI hit, Z = 9.4, RMSD = 2.2 Å for 91 Cα atoms, Figure 2) has been solved in complex with the pili subunit FimH [25], [26] (Figure 2).

### Domain 4 is similar to a chitinase chitin binding domain

The crystal structure of GbpA_D1–3_ lacks the C-terminal domain 4, comprising residues 415–485 (Figure 1). Sequence alignments revealed that this domain shows 26% sequence identity with the C-terminal chitin-binding domain of *S. marcescens* chitinase B (*Sm*ChiB), whose structure is almost devoid of helices and contains 32% β-strands (Supplemental Figure S1 in Text S1, [27]). Indeed, when this GbpA domain (GbpA_D4_) was expressed and purified, circular dichroism experiments showed that it also possesses an essentially all-β secondary structure (Supplemental Figure S1 in Text S1). Interestingly, the sequence alignment suggests that only one (W479) of two aromatic residues (W479 and Y481 [27]) that are thought to be important for *Sm*ChiB-chitin interactions is conserved in GbpA (W463). However, the aromatic residues that form the hydrophobic core of the *Sm*ChiB chitin binding domain (Y470, Y473, W492) are conserved in GbpA. Taken together, it is likely that GbpA possesses an additional C-terminal domain 4, with structural similarity to the *Sm*ChiB C-terminal chitin binding domain.

### GbpA exists as an elongated monomer in solution

To determine the position of domain 4 in the context of the complete GbpA structure, we studied the solution shapes of full length GbpA and GbpA_D1–3_ with Small Angle X-ray Scattering (SAXS). The scattering curves yielded estimated molecular masses for GbpA_fl_ (60±5 kDa) and GbpA_D1–3_ (46±5 kDa) samples that are compatible with their monomeric structures (theoretical masses of 54 kDa and 44 kDa, respectively). This finding is further corroborated by the excluded volumes of GbpA_fl_ (100±10 nm^3^) and GbpA_D1–3_ (80±10 nm^3^), since for sufficiently large globular proteins the hydrated volume in nm^3^ should numerically be about twice the molecular mass in kDa [28]. The experimental radius of gyration R_g_ (3.90±0.05 nm for GbpA_fl_ and 3.55±0.05 nm for GbpA_D1–3_) and maximum diameter D_max_ (13.5±1.0 nm for GbpA_fl_ and 11.5±1.0 nm for GbpA_D1–3_) ) indicate that the proteins behave as extended particles. The solution shapes of GbpA_fl_ and GbpA_D1–3_ were reconstructed *ab initio* using the program DAMMIF [29], with good discrepancy factors (χ = 1.16 for GbpA_fl_ and χ = 1.22 for GbpA_D1–3_). For each protein, ten independent reconstructions produced similar shapes and these were averaged using DAMAVER [30]; the *ab initio* structure is represented in Figure 1D as a wired mesh. To determine the relative orientations of the domains, a model of GbpA_fl_ was generated by rigid-body refinement with the separate GbpA domain structures taken from the domain 1–3 crystal structure (Figure 1B), and the model of domain 4 (Supplemental Figure S1 in Text S1), fitting simultaneously to the experimental SAXS data of GbpA_D1–3_ with χ = 1.41 and of GbpA_fl_ with χ = 1.53. GbpA_fl_ and GbpA_D1–3_ adopt a rod shape with the domains twisted along the long axis (Figure 1D). There are no direct inter-domain interactions, and the surface of each domain appears to be completely exposed to the external environment, suggesting a certain degree of flexibility in the orientation of each domain of GbpA.

### GbpA selectively binds chito-oligosaccharides through domains 1 and 4

**Figure 3:**
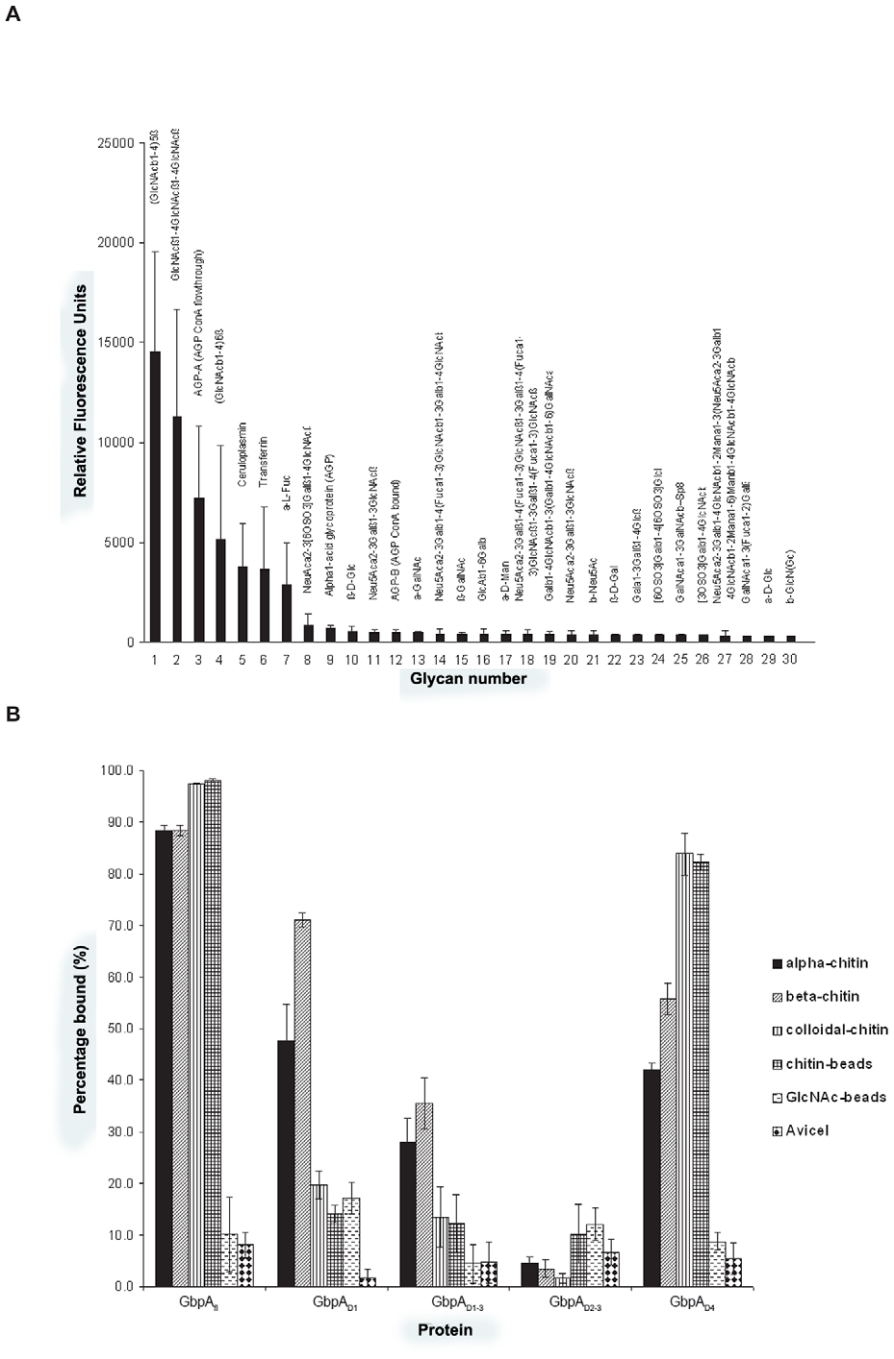
Glycan microarray analysis and chitin binding studies of GbpA. **A.** GbpA_fl_ was screened against an array of glycans. For greater clarity, only those with high signal intensity are presented, and the remaining glycans can be seen in the supplementary section (Supplemental Figure S3 in Text S1). The glycan structures are presented on top of the bar chart, and their abbreviated numbers are shown on the X-axis. The glycans showing the highest binding by GbpA_fl_ are ranked accordingly with penta-GlcNAc (Glycan 1) showing the highest binding by GbpA_fl_ and glucose showing the lowest (Glycan 30). **B.** GbpA_fl_ and truncations were screened for their binding to different forms of chitin/cellulose. Briefly, GbpA_fl_ and various truncations were incubated with chitin or cellulose insoluble substrates. The insoluble substrates were separated by centrifugation, and the amount of protein left in solution (percentage unbound) was measured by Bradford assay. Presented here is the percentage of bound GbpA to the substrate after subtraction of the percentage of the protein unbound in the supernatant.

Previous work has suggested that GbpA binds *N*-acetylglucosamine (GlcNAc) sugars [7]. To investigate whether this extends to GlcNAc-containing glycans, we studied the binding specificity of GbpA by screening a well-established library of mammalian *N*/*O*-linked glycans that also includes a range of linear oligosaccharides [31], [32]. Interestingly, GbpA_fl_ selectively binds chito-oligosaccharides of varying length (Figs. 3A, S5) To quantify chitin binding and identify the domains responsible, direct binding assays were carried out with a range of polysaccharides (Figure 3B). GbpA_D4_ binds to all chitin forms tested with the highest affinity for amorphous forms of chitin (colloidal chitin and chitin beads). GbpA_D1_ shows significant binding only to α- and β-chitin (Figure 3B). GbpA_D2–3_ does not bind chitin. For GbpA_fl_ strong binding to all forms of chitin was observed. None of the GbpA proteins showed binding to GlcNAc-beads or cellulose. Although this is in apparent contrast with a previously published report showing that GbpA-expressing *V. cholerae* bind to GlcNAc beads [7], it could also suggest that additional bacterial factors may be required for the reported GlcNAc binding. Together, these data show that the two terminal chitin-binding domains in GbpA endow the protein with the ability to bind different types of GlcNAc oligomers and polymers.

### Domain 1 of GbpA is required for mucin binding

*V. cholerae* GbpA has been shown to interact with intestinal mucin [13]. To identify the domain(s) involved, GbpA_fl_ and its truncated variants were tested for their ability to bind to mucin, mucin coupled to sepharose beads, intestinal epithelial cells and brush border membranes of the intestine (Table 1). GbpA with a Tyr61Ala (GbpA_(Y61A)_) mutation was also tested to assess whether Tyr61 is important for mucin binding as has been shown for chitin binding in the case of the equivalent Tyr54 in CBP21. Interestingly, only GbpA_fl_, GbpA_(Y61A)_, and GbpA_D1–3_ bind to mucin with µM affinity, further confirmed by ELISA for those domains that tested positive in the initial mucin-binding test. Thus, domain 1 is essential for mucin binding and domain 4 is dispensable for mucin binding. Tyr61 on domain 1 of GbpA is not essential for mucin binding.

**Table 1:** Binding of GbpA_fl_, GbpA_(Y61A)_ and various truncated versions of GbpA to mucin, intestinal epithelial cells (IEC), and brush border membranes (BBM).

GbpA proteins	*K*_d_ (µM) of mucin binding by ELISA	*K*_d_ (µM) of direct mucin binding by Scatchard plot	*K*_d_ (µM) of IEC binding	*K*_d_ (µM) of BBM binding
GbpA_fl_	7.8±1.3	9.4±2.0	11.9±2.1	12.5±0.7
GbpA_(Y61A)_	14.7±0.6	14.1±1.0	18.5±0.6	16.4±0.1
GbpA_D1–3_	13.2±0.7	12.5±1.0	15.4±0.9	13.1±0.3
GbpA_D1_	8.7±0.4	ND	ND	ND
GbpA_D2_	NB*	ND#	ND	ND
GbpA_D3_	76.4±8.3	ND	ND	ND
GbpA_D4_	NB	NB	ND	ND

Values are the means of triplicate determinations from two separate experiments.

*No binding was detectable.

# Not determined.

**Figure 4:**
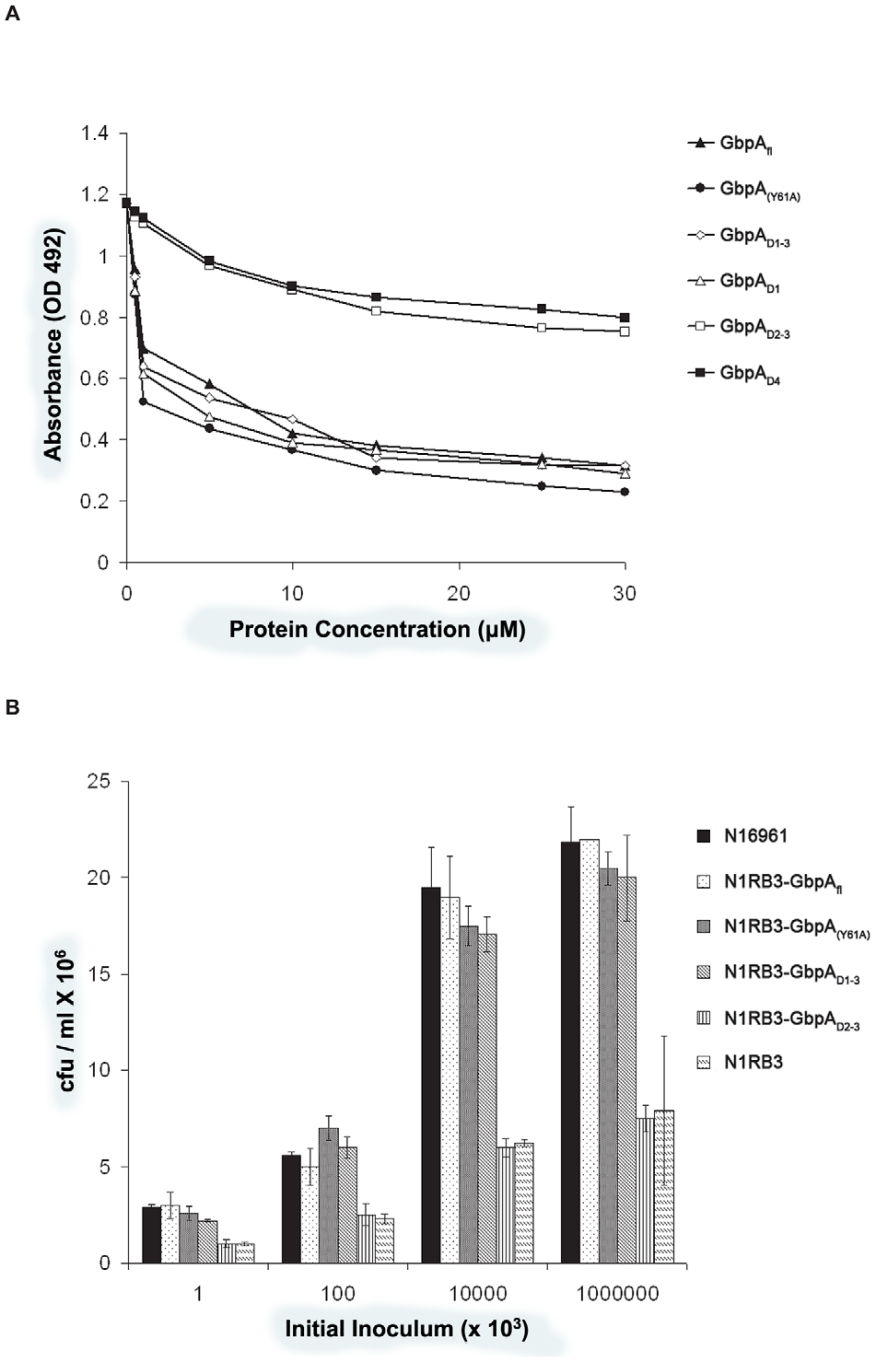
Mucin binding and in vivo colonisation assays of GbpA. **A.** Competition binding assays of wild type *V. cholerae* for surface mucin. Mucin-coated plates were pre-treated with various concentrations of either GbpA_fl_, GbpA_(Y61A)_, or truncated versions of GbpA prior to the addition of labelled wild type *V. cholerae*. The amount of bacteria bound was measured at OD 492. **B.** *In vivo* colonisation studies of wild type and GbpA-complemented *V. cholerae* strains. Mice were orally inoculated with either wild type (N16961), GbpA knock out (N1RB3), or complemented *V. cholerae* strains at varying amount. The intestines of the infected mice were extracted, and the degree of *V. cholerae* colonisation measured. Data represent means ± SEM of at least 4 independent experiments.

To further investigate mucin binding to domain 1, competitive mucin binding assays were carried out with recombinant truncated versions of GbpA and wild type *V. cholerae* (N16961) (Figure 4A). Increasing concentrations of purified GbpA variants added to mucin-coated wells showed varying abilities of the proteins to prevent bacteria from binding the immobilized mucin. GbpA_fl_, GbpA (Y61A), GbpA_D1_ and GbpA_D1–3_ could inhibit binding of N16961 to mucin in a concentration-dependent, saturating manner (Figure 4A). At saturation, GbpA_fl_ and the GbpA_(Y61A)_ mutant inhibited binding of *V. cholerae* to mucin by 83%. Truncated GbpA variants comprising only domains 2, 3, and/or 4 could not inhibit the binding of *V. cholerae* to mucin, in agreement with the direct binding assays.

### GbpA domains 1–3 are essential for intestinal colonization and pathogenesis

In order to understand which domains of GbpA are essential for colonization, we first studied the surface expression of these domains in complemented GbpA knockout *V. cholerae* strain N16961 (N1RB3 or Δ*gbpA*). Immunoblot analysis revealed that the Δ*gbpA* strain complemented with GbpA_fl_, GbpA(Y61A), or GbpA_D1–3_ displays these proteins on the bacterial surface (Supplemental Figure S2 in Text S1). For the strains complemented with GbpA_D1_, GbpA_D2_, GbpA_D2–3_, or GbpA_D4_, no protein could be detected on the bacterial cell surface (Supplemental Figure S2 in Text S1). These results suggest that the presentation of domain 1 on the cell surface is dependent on the presence of domains 2 and 3.

**Figure 5:**
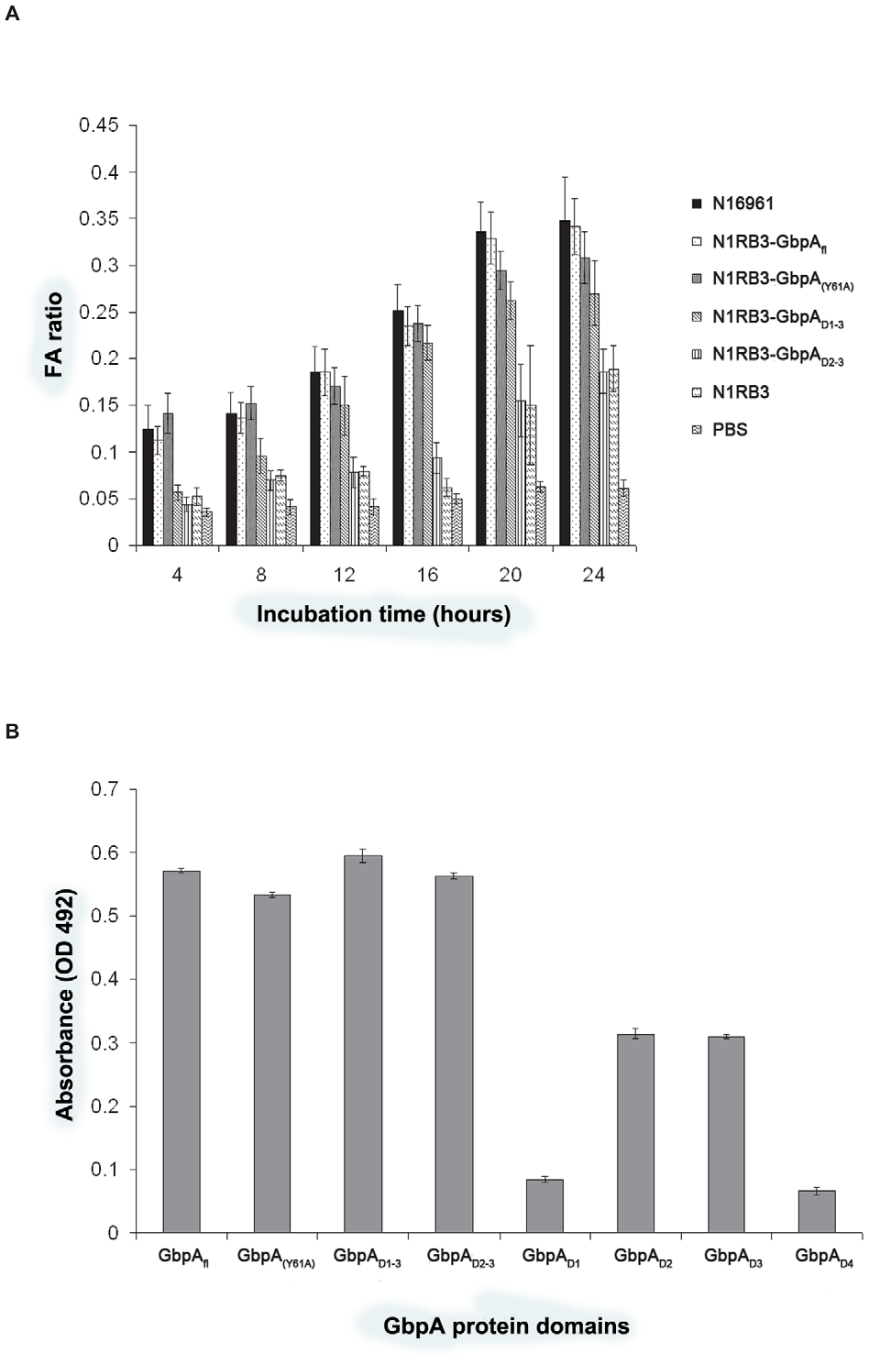
*In vivo* infectivity assays and bacterial surface binding assays of GbpA. **A.** Fluid accumulation assays of mice infected with wild type or GbpA complemented *V. cholerae*. Mice were infected with either wild type (N16961), GbpA knock out (N1RB3), or complemented *V. cholerae* strains. At various times (incubation time) after infection, the mice were sacrificed, and the intestinal weight relative to the whole body without the intestine was measured (FA ratio). Data represent means ± SEM of six independent experiments. **B.** Binding of recombinant GbpA to *V. cholerae* surface. 400 ng of recombinant full length GbpA, or various truncations of GbpA were incubated with 100 µl of GbpA knockout *V. cholerae* strain (N1RB3) at 10^7^ CFU/ml. The amount of each protein that bound to the bacteria was detected using antibodies raised against the recombinant protein.

To probe the role of domains 1–3 in intestinal colonization, the pathogenicity of the complemented strains was analyzed by studying intestinal fluid accumulation and colonization in a mouse model (Figure 4B and Figure 5A). All complemented strains were similar to the wild type strain, with the exception of the GbpA_D2–3_ complemented strain (N1RB3-GbpA_D2–3_), which was affected in its ability to colonize the mice, similar to the Δ*gbpA* N16961 control (N1RB3).

### Domains 2 and 3 of GbpA interact with V. cholerae surface

As shown earlier, GbpA domains 1 and 4 are mainly responsible for chitin, mucin and intestinal epithelium binding. However, GbpA is a secreted protein, and some of the domains must therefore be capable of interacting with *V. cholerae* surface to form a stable host-pathogen interface. To test this, we investigated binding of recombinant full length, mutant and truncated versions of GbpA to the Δ*gbpA* strain (Figure 5B). The results suggest that domains 2 and 3, but not domains 1 or 4, are required for binding to the bacterial surface.

## Discussion

Since the discovery of GbpA, little progress has been made towards an understanding of the molecular properties governing the protein's ability to mediate interactions between the bacteria and the host surfaces, both in the marine environment and the mammalian host. While it has been reported that the protein binds to chitin and intestinal epithelia, the chitin specificity was not probed, nor was it understood how the protein interacts with intestinal epithelial cells. Here we have reported the crystal and solution structures of GbpA, showing an unusual 4-domain elongated structure. Remarkably, the modular domain structure translates to modular interaction properties with different substrates/surfaces. For instance, the first domain has a pseudo-fibronectin type 3 fold that shares considerable similarity with the chitin-binding protein CBP21. Our chitin binding studies showed that this domain, like CBP21, binds to α/β chitin. Furthermore, we were able to demonstrate the interaction of the domain 1 with mucin, a key surface component of intestinal epithelial cells. Thus, the first domain of GbpA harbours the ability to bind to both mucin and certain types of chitin. It is not uncommon that carbohydrate binding modules (CBMs) bind to several substrates or that the same CBM scaffold is used for various binding specificities [20].

For GbpA domains 2 and 3, virtually no sequence similarity to any proteins of experimentally defined function exists. Obtaining the crystal structure of these two domains has given suggestions as to their possible function. Domains 2 and 3 bear some resemblance to proteins such as SFAE and FIMC, chaperones that interact with pili and form part of the chaperone-usher pathway of pili biosynthesis. Both these chaperones reside in the periplasm, but in the case of FimC, reports have shown that the proteins can interact with pili subunits, such as FimH, *in vitro* [33], [34]. Interestingly, our *in vivo* studies show that domains 2 and 3 of GbpA are required for interaction with the bacterial surface. It is also possible that the two domains may facilitate transportation of GbpA to the cell surface via the type II secretion system.

The structure of domain 4 has so far not been obtained, despite numerous attempts to crystallize either the full-length GbpA, this domain alone or as a complex with short chain chitin. The sequence of the domain is distantly related to the chitin binding domain of *Sm*ChiB (Supplemental Figure S1 in Text S1) suggesting a chitin binding function [7]. In our chitin binding assay, we show that domain 4 has greater binding affinity to colloidal chitin and to chitin beads than domain 1. However, domains 1 and 4 together conferred greater binding to chitin of all forms. In contrast to a previously published report [7], no binding of domains 1 and 4 was observed to single GlcNAc residues. Also, domain 4 cannot bind to mucin and is dispensable for *V. cholerae* colonization of intestinal epithelial cells and pathogenesis.

We also attempted to identify the core glycan unit of mucin that GbpA interacts with; however, none were detected in the glycomics screen that includes simple single *O*-linked core structures that are commonly found present on mucins. It is possible that GbpA binding to mucin depends on the glycan-protein linkage, as is not uncommon for other glycan binding proteins [35], or the binding avidity of GbpA for mucin that cannot be simulated on the glycan binding array [36].

In light of our results, we propose the following mechanism for GbpA-mediated bacterial colonization of intestinal epithelial cells. *V. cholerae* constitutively produces GbpA prior to colonization, mainly as a secreted protein. Once in the host intestine, GbpA attaches itself via domain 1 to mucin, thereby marking the surface for *V. cholerae* colonization. Domains 2–3 of the protein then bind to the *V. cholerae* surface, enhancing microcolony formation. In a marine environment, domains 1 and 4 provide GbpA with versatile binding properties to different types of crystalline chitin, allowing the bacterium to attach to, and colonize, a range of crustacea.

## Materials and Methods

### Ethics statement

All animal experiments were conducted following the standard operating procedure as outlined by Committee for the Purpose of Supervision and Control of Experiments on Animals (CPCSEA) Govt. of India. The animal experimental protocol was approved by the Institutional Animal Ethics Committee of National Institute of Cholera and Enteric Diseases (NICED) (Registration No. 68/1999/CPCSEA dated 11-03-1999). New Zealand white rabbits weighing about 2 kg were used for intestinal mucin preparation. 4 to 5 days old BALB/c mice were used for fluid accumulation studies. For intestine harvesting, the animals were euthanized in a CO_2_-chamber. All efforts were made to minimize suffering during euthanasia.

### Cloning and sequencing

The gene coding for full-length *gbpa* (1–485 amino acids, accession number VCA0811) was cloned from genomic DNA of *V. cholerae* into the plasmid pET-22b between the *Nde*I and *Xho*I sites (Novagen). The primers used to amplify the gene were 5′-GCGGAATTCCATATGAAAAAACAACCTAAAATGACCGC-3′ for the forward primer carrying the *Nde*I restriction site, and 5′-CCTCGAGTCATTAACGTTTATCCCACGCCATTTCCC-3′ with the *Xho*I restriction site. From this clone, several truncated versions of GbpA were made either by introducing a stop codon in the original expression vector (pET22b) through the use of the QuickChange mutagenesis kit (Stratagene, La Jolla, CA), or by subcloning into pGEX6P vector (GE Healthcare) through the *Bam*HI and *Xho*I restriction sites. All constructs used are summarised in Supplemental Table S1 in Text S1. Constructs in the pET22b vector (containing the natural transit signalling peptide for secretion) include GbpA comprising of the following amino acids: 1–485 , 1–414, and 1–203. The mature forms of these proteins produced by the bacteria would comprise of the following amino acids: 24–485 (GbpA_fl_), 24–414 (GbpA_D1–3_), and 24–203 (GbpA_D1_). A mutant form of full length GbpA was also generated to introduce a substitution at amino acid position 61 from tyrosine to alanine (GbpA_(Y61A)_). The constructs in the pGEX6P vector consisted of GbpA with N-terminal truncations and covered amino acids 210–315 (GbpA_D2_), 210–414 (GbpA_D2–3_), and 423–485 (GbpA_D4_). The constructs were verified by DNA sequencing (Dundee Sequencing Service).

### Protein production

The constructs of GbpA were transformed into *E. coli* C43. To induce protein expression, the bacteria were grown in LB medium to an OD_600_ of 0.6 before induction with isopropyl-β-D-thiogalactoside (IPTG) at a final concentration of 0.2 mM, and incubation for 16 h at 20°C. Depending on the vector used (pET22b or pGEX6P), two methods were employed to extract the recombinant protein from the bacterial lysate. GbpA_fl_, GbpA_D1–3_, and GbpA_D1_ were prepared from a periplasmic fraction prepared by osmotic shock. The method involved centrifuging (4550× *g*) the cells and then resuspending them in periplasmic buffer (200 mM Tris/HCl pH 7.5, 20% sucrose, 1 mM EDTA and 0.5 mg/ml lysozyme) for 30 minutes at room temperature. The suspension was then cold shocked on ice for 10 minutes, and the periplasmic content isolated by centrifugation at 4000 *g* (4°C). The proteins were further purified by anion exchange chromatography (Amersham, Q sepharose) in 25 mM Tris/HCl (pH 7.5) at a flow rate of 2 ml/min, and eluted with a NaCl gradient (0–500 mM) over a volume of 100 ml. Fractions containing GbpA (GbpA_fl_, GbpA_D1–3_, or GbpA_D1_) were identified by SDS-PAGE and pooled.

GbpA_D2_, GbpA_D2–3_, and GbpA_D4_ were purified from the cytoplasmic fraction of the bacterial cell lysates. The lysis method involves harvesting the induced cells by centrifugation (4550 g), and subsequently resuspending the bacteria in lysis buffer (25 mM Tris/HCl pH 7.5, 150 mM NaCl). Lysozyme (0.1 mg/ml) and DNAse (0.1 mg/l) were added, and the mixture was incubated on ice for 20 minutes. The lysate was then sonicated before centrifugation at 50000 g for 30 minutes. The soluble fraction was collected, passed through a 0.2 µm filter, and incubated with glutathione beads (pre-equilibrated with lysis buffer) for 2 h. Beads containing GbpA were washed with lysis buffer followed by digestion with PreScission protease. Proteins were further purified by gel filtration (26/60 Superdex 200) in lysis buffer.

### Crystallization, structure solution and refinement

Purified GbpA_D1–3_ was concentrated to 25 mg/ml, and used for sitting drop vapor diffusion crystallization experiments using a mother liquor containing 0.2 M Mg(HCO_3_)_2_, 50% (w/v) PEG 3350, 3.33% (w/v) D-sorbitol. For phasing, crystals were soaked with mother liquor containing 20 mM zinc chloride for 12 h. Crystals were cryoprotected with mother liquor containing 5% glycerol (v/v) and then frozen in a nitrogen cryostream. Data were collected on beamline BM14 at the European Synchrotron Radiation Facility and processed with the HKL suite [37] (Supplemental Table S2 in Text S1). Six zinc atoms sites were located by SOLVE, yielding phases to 2.25 Å with an overall figure of merit of 0.30 (Supplemental Table S2 in Text S1) [38]. Combination of the SAD phases with the native amplitudes, solvent flattening and two-fold averaging using DM [39] resulted in an interpretable map. WarpNtrace [40] was used to build a total of 624 (our of 782) residues. The model, excluding 203–208 and 313–318, was completed using Coot [41] interspersed with refinement with REFMAC [42] (Supplemental Table S2 in Text S1). The asymmetric unit contained two monomers, with an RMSD of 0.6 Å. In the interest of simplicity, monomer A was used throughout the data analysis and discussion in this manuscript.

### Small angle X-ray scattering (SAXS)

Synchrotron SAXS data of GbpA were collected according to the standard protocols established at the X33 beam line at the Deutsches Elektronen-Synchrotron DESY (Hamburg, Germany), as detailed in the supplementary material. Briefly, twenty low-resolution models of GbpA_fl_ were built by the program DAMMIF [29] and averaged. A model of GbpA_fl_ was also constructed by rigid body modelling using the program SASREF [43], employing the individual GbpA domain structures.

### Glycan and chitin binding experiments

Recombinant GbpA_fl_ and truncated forms of GbpA were screened for glycan binding against a library of 264 natural and synthetic glycans (100 µM) with amino linkers, and printed onto chemically-modified glass microscope slides. The facility to carry out the experiment (Core H, Printed Array Version 2) was kindly provided by the Consortium of Functional Glycomics (http://www.functionalglycomics.org). The method used to prepare the samples for glycan binding analysis has been reported previously [44]. Six measurements were made and the values averaged (reported here as average Relative Fluorescence Units). Additional results from the screen are available from the supplementary section (Supplemental Figure S2 in Text S1).

### Chitin binding experiments

GbpA_fl_, GbpA_D1–3_, GbpA_D1_, GbpA_D2–3_, and GbpA_D4_ (all 5 µM) in PBS buffer (pH 7.5) were incubated for 24 hours at 21°C with various chitin substrates (5 mg/ml) in a total volume of 300 µl per sample. All samples were run in triplicate, including controls that contained only buffer and substrate. The chitin substrates used were α-chitin (from shrimpshells, Hov-Bio, Tromsø, Norway), β-chitin (from squid pen, France Chitin, Marseille, France), colloidial chitin (made from crab α-chitin from Sigma), chitin-beads (New England BioLabs), GlcNAc-coated agarose beads (Fluka), and Avicel (microcrystalline cellulose, Sigma). The binding experiment was carried out in Eppendorf tubes where protein and substrate were mixed by axial rotation. After incubation, samples were centrifuged for 5 minutes at 25,500× *g*, followed by separation of the pellet (substrate) and supernatant. The protein concentration of the supernatant (unbound protein) was estimated using the Bio-Rad Bradford microassay. The substrate pellets containing bound protein were washed twice with 1.5 ml of PBS, and subsequently boiled for four minutes in 50 µl SDS-PAGE sample buffer. After centrifugation proteins bound to the substrates, now solubilized and denatured were analyzed by SDS-PAGE, using Coomassie protein-stain and methanol/acetic acid (10% v/v) de-stain for visualization of the protein bands.

### Binding studies of GbpA with mucin by ELISA

Rabbit mucin was prepared by intestine scraping as described previously [13]. Briefly, mucin was prepared from by isopyknic ultracentrifugation in cesium chloride [45]. Primary intestinal epithelial cells (IEC) and their brush borders were isolated by previously described methods [46], [47]. IEC were used at a concentration of 2×10^6^ cells/ml and were stimulated for different time points in 5% CO_2_ at 37°C with recombinant GbpA_fl_ and truncations of GbpA. 100 ng of rabbit mucin was coated on the wells of a microtitre plate. The coated plate was kept at 4°C overnight. The unbound mucin was washed with phosphate buffered saline containing 0.5% tween-20 (PBS-T) the next morning. The wells were then blocked with 5% milk in PBS-T for 2 hours. 1–25 µM of recombinant GbpA or its mutant proteins was prepared in PBS, and then applied into the wells in triplicate. After 1.5 hours of incubation, unbound proteins were washed with PBS-T, and anti-GbpA antibody was added to the wells in 1∶250 dilution (prepared in 5% non-fat milk containing PBS-T), prior to incubation for 1.5 hours. Excess antibody was washed in PBS-T, and HRP conjugated anti-mouse antibody (1∶400 dilution) in 5% non-fat milk containing PBS-T was added, and further incubated for 45 minutes. The unbound secondary antibody was washed with PBS-T, and o-phenylenediamine H_2_O_2_ was applied to develop the colour. The absorbance of each well was measured at 492 nm using an automated ELISA reader (Beckman Coulter). The absorbance reading and the maximum binding was assigned the value 1. Data fitting was done using Kyplot version 2.0 Beta15 (32 bit) to obtain the best-fit curves and to obtain the dissociation constant (*Kd*). Values are the means of triplicate determinations from two separate experiments.

### Binding studies of GbpA with immobilized mucin

Rabbit mucin was diluted in binding buffer (0.2 M NaHCO_3_+0.5 M NaCl, pH 8.3) at a protein concentration of 2.6 mg/ml, and then coupled to NHS-activated sepharose resin (GE Healthcare). The unbound mucin was washed with buffer A (0.5 M triethanolamine+0.5 M NaCl pH 8.8) followed by buffer B (0.2 M NaOAc+0.5 M NaCl pH 3.8) following manufacturer's protocol. Different amounts of GbpA_fl_, truncations or its mutants were added in equal volumes to 400 µl of mucin-coupled resin, and the mixtures were incubated for 2 hours at room temperature with shaking. Unbound protein was removed by centrifugation and was measured by protein assay reagent. *K*_d_ was calculated by plotting bound vs. bound/free for each fraction in a Scatchard plot.

### Inhibition of bacterial binding to mucin

100 ng of rabbit mucin was coated on the wells of a microtitre plate as described above. The wells were then blocked with 5% milk in PBS for 2 hours. Different concentrations of GbpA_fl_ and truncations of GbpA were applied to the wells in triplicate. After 1.5 hours of incubation unbound proteins were washed with PBS. *V. cholerae* N16961O1 El Tor was biotinylated as described previously [48]. 1×10^4^ CFU/ml of biotinylated *V. cholerae* were added to each well. After 1.5 hours of incubation, unbound bacteria were washed with PBS, and HRP-conjugated avidin (1∶250 in PBS) was applied to each well. The unbound HRP conjugated avidin was washed with PBS, and o-phenylenediamine H_2_O_2_ was added. The absorbance of each well was measured at 490 nm.

### Transformation of gbpA and its mutant constructs into N1RB3 strain

The *gbpA* knockout strain, N1RB3, was grown overnight as described previously [13]. Briefly, 100 µl of overnight culture were inoculated into 100 ml of LB, and incubated at 37°C to A_600_ = 0.5. The cells were harvested, and washed five times in total 100 ml of sucrose buffer (272 mM sucrose, 1 mM HEPES, 10% glycerol, pH 8). Subsequently, the cells were resuspended in 1/100 of its original volume in buffer containing 272 mM sucrose and 10% glycerol. 50 µL of this cell suspension were used for electroporation with 500 ng of each construct. Pulse conditions were as follows: 2.5 kV voltage, 25 µF capacitance, 200 Ω resistance, and time 3–3.5 ms. After electroporation, cells were resuspended in 950 µL of SOC medium, and grown for 1 hour at 37°C. Then 200 µL of each cell suspension was plated on ampicillin-LB agar, and the plates were incubated for 24 hours at 37°C.

### Isolation of the outer membrane proteins (OMP)

Bacterial cells were harvested from 150 ml of culture of different complemented N1RB3 strains [49]. The cells were washed twice with 0.1 M HEPES (pH 7) before resuspension in 7.5 ml in the same buffer. Then, each preparation was sonicated (intermittent pulse of 15 seconds for 18 pulses) whilst maintaining temperature at 4°C to disrupt the cells. Intact cells were removed by centrifugation at 7000× g for 10 minutes. Each supernatant was further centrifuged at 100000× g for 1 hour, and the pellet was resuspended in 0.5% (w/v) N-laurylsarcosine-Na salt for 15 minutes with gentle agitation. As before, each sample was centrifuged at 100000× g for 1 hour to collect the outer membrane protein as pellet. The pellets were resuspended in 0.1 M HEPES.

### Detection of GbpA on the surface of V. cholerae

Transformation of GbpA_fl_, truncations of GbpA, and its mutant constructs into N1RB3 was performed as reported previously [13]. For ELISA surface expression tests, the complemented strains were grown and fixed, as described previously [50]. The wells were coated with 10^7^ bacterial cells overnight at 4°C. Wells were blocked with 5% non-fat milk in PBS-T. After 2 h of blocking, antibodies against different GbpA truncation mutants were added to the well at a 1∶300 dilution. Excess antibodies were washed off with PBS-T, and HRP-conjugated anti mouse IgG (1∶800) was added. The reaction was developed with o-phenelynediamine and H_2_O_2_. The absorbance was read at 490 nm.

### Intestinal colonization and fluid accumulation assays

Fluid accumulation was assayed as described previously [51]. The assay is a technique used to measure the amount of water and electrolyte accumulation when *V. cholerae* colonizes the intestinal and induces severe diarrhea. Briefly, 4 to 5 days old BALB/c mice were intragastrically inoculated with bacterial inoculum (1×10^5^ CFU) of the appropriate *V. cholerae* strain. Infected mice were sacrificed after 4, 8, 12, 16, 20 and 24 hours post-infection in a CO_2_-chamber. The mice were weighed, and their entire intestines were removed. Each of the separated intestines was weighed, and the FA ratios were calculated as described earlier [51]: FA ratio = intestinal weight/(whole body weight- intestinal weight). PBS-fed mice were used as negative control. All animal experiments were conducted following the guidelines of the Institutional Animal Ethical Committee.

### Binding studies of full length, mutant and truncated GbpA to N1RB3

N1RB3 was grown overnight as described previously [13]. The bacteria were washed twice with PBS. The bacteria were fixed with 0.5% formalin as described previously [50]. After overnight fixation, the bacteria were again washed twice with PBS, and then resuspended in PBS. These bacteria were diluted to 10^8^ CFU/ml (OD_600_ = 0.1), and 100 µL (10^7^ cells) from this suspension was coated in each well, and incubated overnight at 4°C. GbpA_fl_, truncations of GbpA, and its mutants were applied to the wells at a fixed concentration of 400 ng. After 1.5 hours of incubation, polyclonal antibodies against full length, mutant or truncated versions of GbpA were added to the wells at a concentration 1∶300 (v/v) in PBS-T. The amount of recombinant GbpA bound was detected with HRP-conjugated anti-mouse IgG (H+L) at a concentration 1∶800 and o-phenelynediamine+H_2_O_2_. The absorbance at 490 nm was measured in a microplate reader (BIORAD 550 CA).

### Accession numbers

The Protein Data accession number for the coordinates and structure factors of GbpA is 2XWX.

## Supporting Information

Text S1 **Supplementary data.** Three figures (S1-3), two tables (T1-2) and additional Materials and Methods providing additional data to the experiments described in the main text.
(DOC)
