# Supplementary material for: The Vibrio cholerae Colonization Factor GbpA Possesses a Modular Structure that Governs Binding to Different Host Surfaces
Source: PLoS Pathog. 2012 Jan 12;8(1):e1002373. doi: 10.1371/journal.ppat.1002373 (PMC3257281; doi:10.1371/journal.ppat.1002373)
Supplement: Text S1 — Supplementary data. Three figures (S1-3), two tables (T1-2) and additional Materials and Methods providing additional data to the experiments described in the main text. (DOC) [file ppat.1002373.s001.doc]

**Supplemental Table S1.**

Constructs made for recombinant protein production or in vivo expression in GbpA knockout N1RB3 bacterial strain.

| **Protein** | **Abbreviations for complemented bacterial strains** | **Vector** | **Tag at N-terminus** | **Transit sequence (1-23)** | **Purification method** | **Mature protein start residue** | **Mature protein end residue** |
| --- | --- | --- | --- | --- | --- | --- | --- |
| GbpA_fl_ | N1RB3-GbpA_fl_ | pET22b | None | Present | Periplasmic extraction | 24 | 485 |
| GbpA_(Y61A)_ | N1RB3-GbpA_(Y61A)_ | pET22b | None | Present | Periplasmic extraction | 24 | 485 |
| GbpA_D1-3_ | N1RB3-GbpA_D1-3_ | pET22b | None | Present | Periplasmic extraction | 24 | 414 |
| GbpA_D1_ | N1RB3-GbpA_D1_ | pET22b | None | Present | Periplasmic extraction | 24 | 203 |
| GbpA_D2-3_* | N1RB3-GbpA_D2-3_ | pGEX6P | GST | Absent | Cytoplasmic extraction | 210 | 414 |
| GbpA_D2_* | N1RB3-GbpA_D2_ | pGEX6P | GST | Absent | Cytoplasmic extraction | 210 | 315 |
| GbpA_D3_* | N1RB3-GbpA_D3_ | pGEX6P | GST | Absent | Cytoplasmic extraction | 316 | 414 |
| GbpA_D4_ | N1RB3-GbpA_D4_ | pGEX6P | GST | Absent | Cytoplasmic extraction | 423 | 485 |

*For the initial bacterial binding assays a C-terminal FLAG tag was introduced to allow detection of the recombinant protein by anti-FLAG antibodies. For all other experiments no FLAG tag was present in these constructs.

**Supplemental Table S2.**

Data collection and refinement statistics. The values in parenthesis refer to the highest resolution shell.

|  | **GbpA_D1-3_  native** | **GbpA_D1-3_  Zn-SAD** |
| --- | --- | --- |
|  | |  |
| Wavelength (Å) | 0.933 | 1.281 |
| Resolution (Å) | 20.0 - 1.80 (1.86-1.80) | 20.00 - 2.25 (2.33-2.25) |
| Space group | *P*21 | *P*21 |
| Unit cell (Å,^o^) |  |  |
| *a* | 60.0 | 55.3 |
| *b* | 120.2 | 119.2 |
| *c* | 67.2 | 66.9 |
|   β | 108.4 | 108.2 |
| Reflections |  |  |
| Observed | 224799 (19567) | 230904 (1922) |
| Unique | 73728 (7005) | 32995 (470) |
| Redundancy | 3.0 (2.8) | 7.0 (4.1) |
| R_merge_ | 0.048 (0.330) | 0.107 (0.422) |
| I/σI | 14.4 (3.0) | 12.3 (2.9) |
| Completeness (%) | 95.2 (91.6) | 83.7 (11.9) |
| R_cryst_ (%) | 20.7 |  |
| R_free_ (%) | 24.6 |  |
| Total number of atoms | |  |
| Protein | 6014 (770 residues) |  |
| Water | 630 |  |
| <*B*> Protein (Å^2^) | 37.5 |  |
| <*B*> Water (Å^2^) | 45.6 |  |
| RMSD from ideal geometry | | |
| Bond lengths (Å) | 0.008 |  |
| Bond angles (^o^) | 1.124 |  |
| Ramachandran plot |  |  |
| Preferred (%) | 96.9 |  |
| Allowed (%) | 3.0 |  |
| Outliers (%) | 0.1 |  |

**Supplemental Figure S1.**

Secondary structure prediction of GbpA_D4_.

**A)** ClustalW sequence alignment between GbpA_D4_ and the chitin binding domain of *Serratia marcescens* chitinase B (*Sm*ChiB). The numbering above the sequence denotes the residue numbers in GbpA. The secondary structure below the sequence refers to the published *Sm*ChiB structure (1E6P.pdb).

**B)** Recombinant GbpA_D4_ (amino acids 423-485) was analysed by CD spectroscopy (Y axis units in: [θ] x 10^-3^ (deg.cm^2^.dmol^-1^); X axis units in: wavelength (nm)). The spectrum was deconvoluted using the program CONTINLL, and the secondary structure composition is shown besides the chart.

**C)** The secondary structure composition from the molecular structure of the chitin binding domain (amino acids 450 - 498) of *Sm*ChiB (ribbon diagram and molecular surface) was calculated, and the values depicted beside the structure.

**A) Sequence alignment between GbpA_D4_ and *Sm*ChiB**


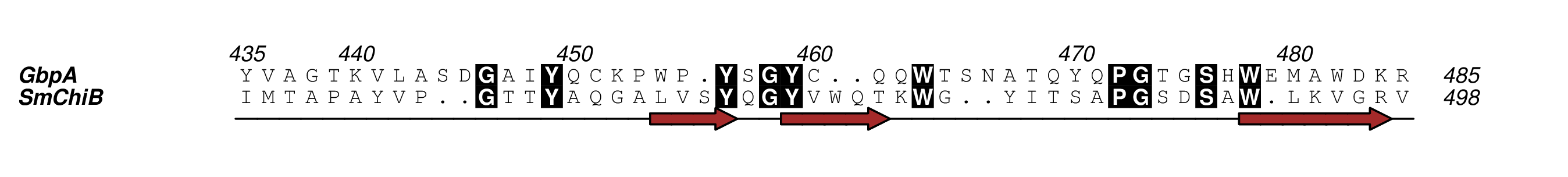


**B) GbpA_D4_ CD spectrum**

**C) *Sm*ChiB chitin binding domain**

**A)**

Helix: 5 %

Sheet: 32 %

Turns: 63%

Helix: 0 %

Sheet: 32 %

Turns: 68%

**Supplemental Figure S2.**

Expression of GbpA on the surface of *Vibrio cholerae*.

**A)** The expression of GbpA on the surface of wild-type *Vibrio cholerae* (N16961), and GbpA knockout *Vibrio cholerae* strain (N1RB3) was detected by using antibodies raised against recombinant GbpA. The amount of antibodies against GbpA bound to the surface was measured at OD_490_ as relative absorbance. Similarly, surface expression of GbpA was measured for the complemented GbpA knockout strains.

**B)** Western blot for the presence of GbpA on the surface of the bacteria after membrane extraction. The complemented strains are as follows: N1RB3-GbpA_D2-3_ strain was complemented with GbpA_D2-3_, N1RB3-GbpA_D1_ strain was complemented with GbpA_D1_, N1RB3-GbpA_D4_ strain was complemented with GbpA_D4_, N1RB3-GbpA_D2-3_ strain was complemented with GbpA_D2-3_, N1RB3-GbpA_fl_ strain was complemented with GbpA_fl_, N1RB3-GbpA(Y61A) strain was complemented with GbpA(Y61A), and N1RB3-GbpA_D1-3_ was complemented with GbpA_D1-3_.

**A)**

**
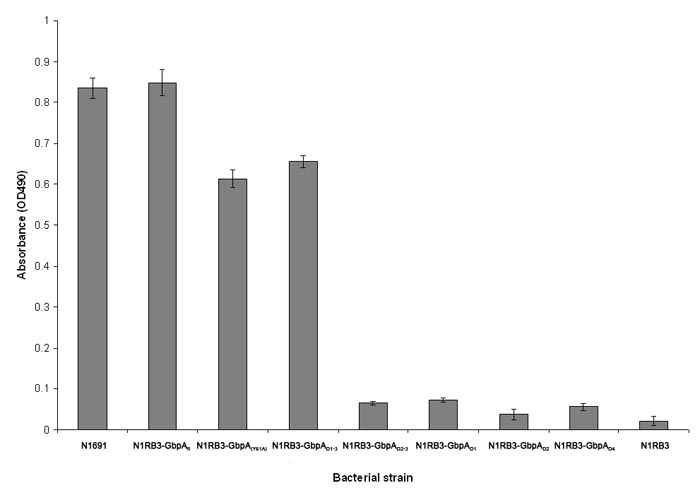
**

Domains of GbpA expressed in N1RB3

**B)**

**B)**

**Supplemental Figure S3.**

Glycan screen of GbpA_fl_. Full length GbpA was screened against 264 glycans that were attached to a surface of varying linkage lengths (Sp) (see Materials and Methods). The affinity of GbpA_fl_ for various glycans was ranked accordingly; glycans ranked at the top are those showing the highest affinity for GbpA_fl_, whilst those at the bottom shows the lowest binding affinity. The data is also available at the Consortium of Functional Glycomics ([http://www.functionalglycomics.org](http://www.functionalglycomics.org/)).

| **Identifier** | **Glycan** | **Relative Fluorescence Units** | **Standard Deviation** |
| --- | --- | --- | --- |
| 1 | (GlcNAcb1-4)_5_β-Sp8 | 14538.16968 | 4994.035735 |
| 2 | GlcNAcβ1-4GlcNAcβ1-4GlcNAcβ–Sp8 | 11305.76708 | 5367.771809 |
| 3 | AGP-A (AGP ConA flowthrough) | 7223.9115 | 3614.95497 |
| 4 | (GlcNAcb1-4)_6_β-Sp8 | 5108.803375 | 4744.241808 |
| 5 | Ceruloplasmin | 3771.39265 | 2151.515471 |
| 6 | Transferrin | 3619.56275 | 3165.024343 |
| 7 | α-L-Fuc–Sp8 | 2847.044025 | 2156.169556 |
| 8 | NeuAcα2-3[6OSO3]Galβ1-4GlcNAcβ–Sp8 | 852.048875 | 546.4843517 |
| 9 | Alpha1-acid glycoprotein (AGP) | 677.346125 | 158.2079267 |
| 10 | β-D-Glc–Sp8 | 489.991225 | 280.2979906 |
| 11 | Neu5Acα2-3Galβ1-3GlcNAcβ–Sp0 | 485.982975 | 103.9375961 |
| 12 | AGP-B (AGP ConA bound) | 475.216225 | 118.749175 |
| 13 | α-GalNAc–Sp8 | 454.9387 | 58.18023764 |
| 14 | Neu5Aca2-3Galb1-4(Fuca1-3)GlcNAcb1-3Galb1-4GlcNAcb-Sp8 | 435.757975 | 220.1543082 |
| 15 | β-GalNAc–Sp8 | 418.515475 | 37.54788901 |
| 16 | GlcAb1-6Galb-Sp8 | 416.761925 | 246.7552853 |
| 17 | α-D-Man–Sp8 | 416.4442375 | 124.7059147 |
| 18 | Neu5Acα2-3Galβ1-4(Fucα1-3)GlcNAcβ1-3Galβ1-4(Fucα1-3)GlcNAcβ1-3Galβ1-4(Fucα1-3)GlcNAcβ–Sp0 | 416.03735 | 202.54073 |
| 19 | Galb1-4GlcNAcb1-3(Galb1-4GlcNAcb1-6)GalNAca-Sp8 | 406.49665 | 114.5800051 |
| 20 | Neu5Acα2-3Galβ1-3GlcNAcβ–Sp8 | 390.64735 | 188.3352917 |
| 21 | b-Neu5Ac-Sp8 | 379.878175 | 167.5672927 |
| 22 | β-D-Gal–Sp8 | 373.1115 | 42.45707782 |
| 23 | Galα1-3Galβ1-4Glcβ–Sp0 | 365.752275 | 38.6789594 |
| 24 | [6OSO3]Galb1-4[6OSO3]Glcb-Sp8 | 354.96605 | 61.08107936 |
| 25 | GalNAcα1-3GalNAcb–Sp8 | 353.98475 | 81.01055668 |
| 26 | [3OSO3]Galb1-4GlcNAcb-Sp8 | 350.63795 | 44.38436856 |
| 27 | Neu5Aca2-3Galb1-4GlcNAcb1-2Mana1-3(Neu5Aca2-3Galb1-4GlcNAcb1-2Mana1-6)Manb1-4GlcNAcb1-4GlcNAcb-Sp8 | 348.31 | 207.96521 |
| 28 | GalNAcα1-3(Fucα1-2)Galβ–Sp8 | 347.906375 | 138.8043333 |
| 29 | α-D-Glc–Sp8 | 344.6573 | 60.7936256 |
| 30 | b-GlcN(Gc)-Sp8 | 338.4937 | 49.15151834 |
| 31 | [4OSO3]Galb1-4GlcNAcb-Sp8 | 331.324475 | 56.09557504 |
| 32 | Fucα1-2Galβ1-3GalNAcβ1-4(Neu5Acα2-3)Galβ1-4Glcβ-Sp9 | 329.8779 | 47.26180257 |
| 33 | Galβ1-3(Fucα1-4)GlcNAc–Sp8 | 326.410625 | 66.85858162 |
| 34 | Neu5Aca2-3Galb1-4GlcNAcb1-2Mana1-3(Neu5Aca2-3Galb1-4GlcNAcb1-2Mana1-6)Manb1-4GlcNAcb1-4GlcNAcb-Gly | 322.360725 | 161.4458804 |
| 35 | Galb1-4GalNAcb1-3(Fuca1-2)Galb1-4GlcNAcb-Sp8 | 322.075525 | 102.8128421 |
| 36 | Fibrinogen | 316.540525 | 49.10537912 |
| 37 | Fuca1-2Galb1-4(Fuca1-3)GlcNAcb1-3Galb1-4(Fuca1-3)GlcNAcb1-3Galb1-4(Fuca1-3)GlcNAcb-Sp0 | 312.465125 | 5.12694431 |
| 38 | β-GlcNAc–Sp8 | 311.465575 | 63.36376011 |
| 39 | [3OSO3]Galβ1-4GlcNAcβ–Sp0 | 309.699 | 96.80133957 |
| 40 | Glcb1-4Glcb-Sp8 | 309.302425 | 49.11078158 |
| 41 | Neu5Acα2-3Galβ1-4GlcNAcβ1-3Galβ1-4GlcNAcβ1-3Galβ1-4GlcNAcβ–Sp0 | 308.43695 | 173.4340088 |
| 42 | Gala1-4(Fuca1-2)Galb1-4GlcNAcb-Sp8 | 306.985825 | 27.60059326 |
| 43 | [3OSO3]Galβ1-4(6OSO3)Glcβ–Sp0 | 304.3101 | 62.69021962 |
| 44 | Fucb1-3GlcNAcb-Sp8 | 302.80815 | 71.24510715 |
| 45 | GalNAcα1-3(Fucα1-2)Galβ1-4GlcNAcβ–Sp8 | 300.1305 | 67.88908894 |
| 46 | Fuca1-2Galb1-3GalNAcb1-3Gala1-4Galb1-4Glcb-Sp9 | 298.91815 | 193.988305 |
| 47 | GalNAca1-3(Fuca1-2)Galb1-4(Fuca1-3)GlcNAcb-Sp0 | 298.76945 | 72.03735993 |
| 48 | [3OSO3]Galβ1-4(Fucα1-3)GlcNAcβ–Sp8 | 295.992625 | 89.89933436 |
| 49 | [3OSO3][6OSO3]Galb1-4[6OSO3]GlcNAcb-Sp0 | 292.086275 | 53.09066582 |
| 50 | Galβ1-3GlcNAcβ1-3Galβ1-4Glcβ–Sp10 | 291.9378 | 64.97258862 |
| 51 | Galb1-4GlcNAcb1-2Mana1-3(Galb1-4GlcNAcb1-2Mana1-6)Manb1-4GlcNAcb1-4GlcNAcb-Gly | 291.6353 | 147.5636743 |
| 52 | β-GlcNAc–Sp0 | 291.5403 | 43.14934058 |
| 53 | Galα1-3Galβ1-4(Fucα1-3)GlcNAcβ–Sp8 | 288.93525 | 29.77812722 |
| 54 | GalNAcβ1-4GlcNAcβ–Sp8 | 288.184775 | 60.69145463 |
| 55 | Galα1-4Galβ1-4GlcNAcβ–Sp0 | 287.81475 | 43.69886789 |
| 56 | Mana1-3(Mana1-6)Manb1-4GlcNAcb1-4GlcNAcb-Gly | 287.03295 | 89.76959395 |
| 57 | GlcNAcb1-2Mana1-3(GlcNAcb1-2Mana1-6)Manb1-4GlcNAcb1-4GlcNAcb-Gly | 285.94645 | 13.0935514 |
| 58 | Galβ1-4[6OSO3]Glcβ–Sp0 | 284.3393 | 49.96927577 |
| 59 | Fuca1-2GlcNAcb-Sp8 | 280.28565 | 91.40155739 |
| 60 | Gala1-3(Gala1-4)Galb1-4GlcNAcb-Sp8 | 279.41825 | 53.1844221 |
| 61 | GalNAca1-3(Fuca1-2)Galb1-4GlcNAcb-Sp0 | 279.0566 | 43.98411028 |
| 62 | Fuca1-2Galb1-4(Fuca1-3)GlcNAcb1-3Galb1-4(Fuca1-3)GlcNAcb-Sp0 | 276.72695 | 48.53556655 |
| 63 | Fucα1-2Galβ1-3GlcNAcβ1-3Galβ1-4Glcβ–Sp8 | 271.739325 | 13.01731819 |
| 64 | Galβ1-3GlcNAcβ–Sp8 | 271.441025 | 19.14303824 |
| 65 | [3OSO3]Galβ1-3(Fucα1-4)GlcNAcβ–Sp8 | 268.958025 | 58.41507163 |
| 66 | Neu5Aca2-8Neu5Aca2-8Neu5Aca2-3(GalNAcb1-4)Galb1-4Glcb-Sp0 | 268.06625 | 99.9823341 |
| 67 | Galb1-3(Fuca1-4)GlcNAcb1-3Galb1-4GlcNAcb-Sp0 | 266.805175 | 38.59152211 |
| 68 | Fucα1-2Galβ1-3(Fucα1-4)GlcNAcβ–Sp8 | 264.913375 | 69.0688783 |
| 69 | Galβ1-4(Fucα1-3)GlcNAcβ1-4Galβ1-4(Fucα1-3)GlcNAcβ1-4Galβ1-4(Fucα1-3)GlcNAcβ–Sp0 | 263.0621 | 108.6202662 |
| 70 | Fuca1-2Galb1-3GalNAcb1-4(Neu5Aca2-3)Galb1-4Glcb-Sp0 | 262.465325 | 32.17682651 |
| 71 | α-D-Gal–Sp8 | 258.6452 | 45.31386361 |
| 72 | 6-H_2_PO_3_Manα–Sp8 | 258.587125 | 79.04061845 |
| 73 | Galβ1-3(Galβ1-4GlcNAcβ1-6)GalNAcα-Sp8 | 254.857725 | 61.5594346 |
| 74 | [3OSO3]Galβ1-3GlcNAcβ–Sp8 | 254.5361 | 68.07135417 |
| 75 | [6OSO3]Galβ1-4GlcNAcβ–Sp8 | 252.9274 | 91.21692133 |
| 76 | Gala1-3(Fuca1-2)Galb1-4(Fuca1-3)GlcNAcb-Sp0 | 251.1281 | 60.05315362 |
| 77 | [3OSO3]Galb1-4[6OSO3]GlcNAcb-Sp8 | 247.9618 | 16.71078873 |
| 78 | [3OSO3]Galβ1-4(6OSO3)Glcβ–Sp8 | 247.02985 | 69.9543539 |
| 79 | GlcNAcb1-4(GlcNAcb1-6)GalNAca-Sp8 | 246.428375 | 92.50595551 |
| 80 | GalNAca1-3(Fuca1-2)Galb1-3GlcNAcb-Sp0 | 244.2769 | 40.32655774 |
| 81 | Galβ1-4[6OSO3]Glcβ–Sp8 | 243.17275 | 28.75907122 |
| 82 | Galα1-4Galβ1-4GlcNAcβ–Sp8 | 241.973375 | 47.98523143 |
| 83 | Galβ1-2Galβ–Sp8 | 241.22015 | 51.2132207 |
| 84 | [3OSO3]Galβ–Sp8 | 240.80905 | 104.9558634 |
| 85 | GalNAcβ1-4GlcNAcβ–Sp0 | 239.95985 | 41.64195083 |
| 86 | Galα1-3Galβ–Sp8 | 239.4343 | 57.6353766 |
| 87 | Fucα1-3GlcNAcβ–Sp8 | 239.343525 | 83.32438011 |
| 88 | [3OSO3][6OSO3]Galb1-4GlcNAcb-Sp0 | 237.5585 | 58.2716326 |
| 89 | Fucα1-2Galβ1-4(Fucα1-3)GlcNAcβ–Sp8 | 236.75715 | 50.81329486 |
| 90 | α-L-Rhα–Sp8 | 234.792875 | 50.07096306 |
| 91 | GlcNAcb1-3Galb-Sp8 | 234.333175 | 10.63242469 |
| 92 | GlcNAcb1-6Galb1-4GlcNAcb-Sp8 | 233.688925 | 37.20611701 |
| 93 | 9NAcNeu5Aca2-6Galb1-4GlcNAcb-Sp8 | 233.55495 | 50.01702743 |
| 94 | Mana1-2Mana1-3Mana-Sp9 | 232.1604 | 51.32101041 |
| 95 | Neu5Aca2-3Galb-Sp8 | 230.2653 | 39.0471744 |
| 96 | Galα1-4GlcNAcb–Sp8 | 228.6471 | 45.51529386 |
| 97 | NeuAca2-3Galb1-3(Fuca1-4)GlcNAcb1-3Galb1-4(Fuca1-3)GlcNAcb-Sp0 | 227.2762 | 45.45002385 |
| 98 | Galβ1-3GalNAcα-Sp8 | 226.4786 | 41.68112253 |
| 99 | [4OSO3][6OSO3]Galb1-4GlcNAcb-Sp0 | 225.531925 | 24.60197839 |
| 100 | Manα1-3(Manα1-6)Manα–Sp9 | 225.267125 | 81.82630819 |
| 101 | Galα1-3(Fucα1-2)Galβ–Sp8 | 224.4062 | 51.23161157 |
| 102 | [3OSO3]Galb1-4Glcb-Sp8 | 223.96445 | 82.76601417 |
| 103 | Neu5Aca2-6Galb1-4GlcNAcb1-3Galb1-4(Fuca1-3)GlcNAcb1-3Galb1-4(Fuca1-3)GlcNAcb-Sp0 | 223.424375 | 42.95374797 |
| 104 | GalNAcb1-4(Fuca1-3)GlcNAcb-Sp0 | 220.927375 | 51.47047252 |
| 105 | Gala1-3(Fuca1-2)Galb1-3GlcNAcb-Sp0 | 219.183175 | 69.83216677 |
| 106 | Neu5Aca2-3Galb1-3GalNAca-Sp8 | 218.85295 | 82.27939141 |
| 107 | NeuAca2-3(NeuAca2-3Galb1-3GalNAcb1-4)Galb1-4Glcb-Sp0 | 218.55875 | 93.07878093 |
| 108 | Man5-9mix-Asn | 217.4442 | 82.34001199 |
| 109 | Galβ1-3GlcNAcβ–Sp0 | 217.061775 | 87.42987014 |
| 110 | Glcα1-6Glcα1-6Glcβ-Sp8 | 216.802675 | 159.5978867 |
| 111 | GalNAcα1-3Galb–Sp8 | 216.30845 | 34.62291812 |
| 112 | Gala1-3(Fuca1-2)Galb1-4GlcNAc-Sp0 | 216.206225 | 83.6931245 |
| 113 | [6OSO3]Galβ1-4Glcβ–Sp0 | 215.5667 | 34.31806467 |
| 114 | GlcNAcβ1-3Galβ1-4Glcβ–Sp0 | 214.87965 | 19.63501427 |
| 115 | Neu5Acb2-6Galb1-4GlcNAcb-Sp8 | 212.992125 | 29.4420367 |
| 116 | Galβ1-4GlcNAcβ1-3Galβ1-4Glcβ–Sp8 | 212.790325 | 62.62875456 |
| 117 | Fuca1-2Galb1-4GlcNAcb1-3Galb1-4GlcNAcb1-3Galb1-4GlcNAcb-Sp0 | 210.95765 | 87.77343763 |
| 118 | Neu5Gcα–Sp8 | 210.61245 | 61.44127636 |
| 119 | Fucα1-2Galβ1-4Glcβ–Sp0 | 210.229875 | 56.9238861 |
| 120 | [6OSO3]GlcNAcβ–Sp8 | 209.6586 | 73.38863046 |
| 121 | Manα1-6(Manα1-3)Manα1-6(Manα2Manα1-3)Manβ1-4GlcNAcβ1-4GlcNAcβ-Asn | 208.37155 | 43.83543928 |
| 122 | Mana1-2Mana1-2Mana1-3Mana-Sp9 | 207.124375 | 36.02350047 |
| 123 | GalNAcb1-3Gala1-4Galb1-4GlcNAcb-Sp0 | 205.7707 | 46.14864067 |
| 124 | Fucα1-2Galβ1-3GlcNAcβ–Sp0 | 204.517225 | 46.88889728 |
| 125 | Fucα1-2Galβ1-3GlcNAcβ–Sp8 | 202.53395 | 51.86782196 |
| 126 | GlcNAcb1-4Galb1-4GlcNAcb-Sp8 | 201.5099 | 23.05128759 |
| 127 | GlcAa-Sp8 | 200.520025 | 13.67589949 |
| 128 | Galα1-2Galβ–Sp8 | 200.4525 | 56.24708698 |
| 129 | 9NAcNeu5Aca-Sp8 | 199.928525 | 42.99783771 |
| 130 | Fuca1-2Galb1-3GalNAcb1-3Gala-Sp9 | 199.904025 | 110.9856369 |
| 131 | GlcAb1-3Galb-Sp8 | 196.725125 | 107.3061265 |
| 132 | Neu5Acα2-3Galβ1-4(Fucα1-3)GlcNAcβ–Sp8 | 196.6958 | 49.70002801 |
| 133 | GlcNAca1-6Galb1-4GlcNAcb-Sp8 | 196.0297 | 70.90022245 |
| 134 | Gala1-3(Fuca1-2)Galb1-4Glcb-Sp0 | 194.854325 | 48.68987542 |
| 135 | Neu5Aca2-6Galb1-4[6OSO3]GlcNAcb-Sp8 | 194.122425 | 45.53065751 |
| 136 | (GlcNAcb1-3(GlcNAcb1-6)GlcNAcb1-4)GlcNAc-Sp8 | 192.964225 | 62.35886399 |
| 137 | Neu5Aca2-3Galb1-3[6OSO3]GalNAca-Sp8 | 191.7273 | 39.56821011 |
| 138 | Gala1-3GalNAca-Sp8 | 191.134175 | 46.72714884 |
| 139 | Gala1-6Glcb-Sp8 | 190.801475 | 30.2287917 |
| 140 | Manb1-4GlcNAcb-Sp0 | 190.138225 | 49.8189316 |
| 141 | α-Neu5Ac–Sp8 | 189.792275 | 93.22347266 |
| 142 | Neu5Aca2-3Galb1-4(Fuca1-3)GlcNAcb1-3Galb-Sp8 | 188.0012 | 99.47465728 |
| 143 | Galβ1-3GalNAcβ1-4Galβ1-4Glcβ–Sp8 | 187.880825 | 37.06844366 |
| 144 | GalNAca1-4(Fuca1-2)Galb1-4GlcNAcb-Sp8 | 186.660575 | 66.83834762 |
| 145 | Manα1-2Manα1-6(Manα1-3)Manα1-6(Manα2Manα2Manα1-3)Manβ1-4GlcNAcβ1-4GlcNAcβ-Asn | 182.944875 | 70.96397381 |
| 146 | NeuAca2-3Galb1-3GalNAcb1-3Gala1-4Galb1-4Glcb-Sp0 | 182.536925 | 50.3539773 |
| 147 | Fucα1-2Galβ1-4GlcNAcβ1-3Galβ1-4GlcNAc–Sp0 | 179.64775 | 29.29248923 |
| 148 | Glcα1-4Glca–Sp8 | 178.6549 | 44.0327402 |
| 149 | Galb1-4GlcNAcb1-3Galb1-4(Fuca1-3)GlcNAcb1-3Galb1-4(Fuca1-3)GlcNAcb-Sp0 | 178.3966 | 48.39684489 |
| 150 | Galβ1-4(Fucα1-3)GlcNAcb–Sp8 | 178.153675 | 23.76869872 |
| 151 | Fucα1-2Galβ1-3GalNAcα–Sp8 | 174.13055 | 45.39964231 |
| 152 | Fucα1-2Galβ1-4GlcNAcβ–Sp8 | 173.113 | 15.90059264 |
| 153 | GalNAcb1-3GalNAca–Sp8 | 171.448425 | 23.56217877 |
| 154 | GalNAcb1-3(Fuca1-2)Galb-Sp8 | 171.372025 | 30.57782219 |
| 155 | Galβ1-3Galβ–Sp8 | 170.6205 | 50.96487378 |
| 156 | GlcAb-Sp8 | 170.172725 | 72.05746257 |
| 157 | Galα1-4Galβ1-4Glcβ–Sp0 | 170.0762 | 24.81973678 |
| 158 | Gala1-3Galb1-3GlcNAcb-Sp0 | 169.8854 | 33.7291004 |
| 159 | Neu5Aca2-3(Galb1-3GalNAcb1-4)Galb1-4Glcb-Sp0 | 169.7929 | 37.10604626 |
| 160 | Galb1-4(Fuca1-3)GlcNAcb1-4Galb1-4(Fuca1-3)GlcNAcb-Sp0 | 167.050525 | 32.71149622 |
| 161 | [6OSO3]Galβ1-4Glcβ–Sp8 | 164.0692 | 11.84732543 |
| 162 | Neu5Acβ2-6(Galβ1-3)GalNAcα–Sp8 | 163.487325 | 89.00697975 |
| 163 | α-L-Fuc–Sp9 | 163.453025 | 64.12277914 |
| 164 | Galb1-4GalNAca1-3(Fuca1-2)Galb1-4GlcNAcb-Sp8 | 161.5932 | 77.70288592 |
| 165 | Galb1-3GalNAcb1-4(Neu5Aca2-3)Galb1-4Glcb-Sp0 | 161.330575 | 37.42646551 |
| 166 | Neu5Acα2-3(Neu5Acα2-6)GalNAcα–Sp8 | 160.0469 | 32.71684104 |
| 167 | Mana1-6(Mana1-2Mana1-3)Mana1-6(Manα2Manα1-3)Manb1-4GlcNAcb1-4GlcNAcb-Asn | 159.3597 | 23.63527281 |
| 168 | Neu5Aca2-6GalNAcb1-4GlcNAcb-Sp0 | 158.4542 | 48.496061 |
| 169 | Neu5Acβ1-6GalNAcα–Sp8 | 157.9062 | 20.95992709 |
| 170 | Galβ1-4GlcNAcβ1-3Galβ1-4GlcNAcβ–Sp0 | 157.4401 | 35.34505324 |
| 171 | Galb1-3(Neu5Aca2-6)GlcNAcb1-4Galb1-4Glcb-Sp10 | 156.86675 | 54.9560924 |
| 172 | Neu5Gcα2-6Galβ1-4GlcNAcβ–Sp0 | 156.796325 | 66.42458025 |
| 173 | Galβ1-3GalNAcβ–Sp8 | 152.816675 | 30.78400835 |
| 174 | KDNα2-3Galβ1-4GlcNAcβ–Sp0 | 152.532825 | 35.77253346 |
| 175 | Neu5Aca2-3(GalNAcb1-4)Galb1-4GlcNAcb-Sp0 | 152.36265 | 66.28860424 |
| 176 | Mana1-3(Mana1-2Mana1-2Mana1-6)Mana-Sp9 | 151.848975 | 18.78113981 |
| 177 | NeuAca2-3Galb1-3GlcNAcb1-3Galb1-4GlcNAcb-Sp0 | 151.5774 | 32.48215564 |
| 178 | Galb1-3(Neu5Acb2-6)GalNAca-Sp8 | 151.4897 | 21.98686962 |
| 179 | Neu5Acα2-3Galβ1-4GlcNAcβ–Sp0 | 150.799775 | 26.91132623 |
| 180 | Galβ1-4Glcβ–Sp8 | 149.844025 | 10.97055642 |
| 181 | Galb1-3(Fuca1-4)GlcNAcb1-3Galb1-4(Fuca1-3)GlcNAcb-Sp0 | 143.909925 | 41.44353327 |
| 182 | Galb1-3(Neu5Aca2-6)GalNAca-Sp8 | 142.330175 | 49.06343012 |
| 183 | GlcNAca1-3Galb1-4GlcNAcb-Sp8 | 141.72455 | 19.26009033 |
| 184 | Neu5Acα2-6Galβ1-4Glcβ–Sp0 | 139.104175 | 50.02905911 |
| 185 | Neu5Acα2-3Galβ1-4Glcβ–Sp0 | 139.00355 | 44.18752412 |
| 186 | GlcNAcβ1-3(GlcNAcβ1-6)GalNAcα–Sp8 | 138.744 | 58.78193201 |
| 187 | Galα1-3Galβ1-4GlcNAcβ–Sp8 | 138.15175 | 40.50757113 |
| 188 | Neu5Acα2-6Galβ–Sp8 | 137.7008 | 38.50796651 |
| 189 | Neu5Acα2-3Galβ1-3(Neu5Acα2-6)GalNAcα–Sp8 | 137.1473 | 13.79128869 |
| 190 | [3OSO3]Galβ1-3GalNAcα–Sp8 | 135.611675 | 47.55849567 |
| 191 | Neu5Aca2-8Neu5Acα2-3(GalNAcβ1-4)Galβ1-4Glcβ–Sp0 | 135.231425 | 30.83566211 |
| 192 | Galb1-3GalNAcb1-3Gala1-4Galb1-4Glcb-Sp0 | 135.106625 | 48.34506762 |
| 193 | Neu5Gca2-3Galb1-3GlcNAcb-Sp0 | 132.3971 | 31.74368262 |
| 194 | GlcNAcβ1-3Galβ1-4GlcNAcβ–Sp0 | 130.730125 | 52.14584269 |
| 195 | Neu5Aca2-3Galb1-4[6OSO3]GlcNAcb-Sp8 | 130.719975 | 45.31174129 |
| 196 | Galβ1-4GlcNAcβ1-6GalNAcα–Sp8 | 130.390825 | 29.89710002 |
| 197 | Neu5Acα2-8Neu5Acα2-3Galβ1-4Glcβ–Sp0 | 129.258675 | 10.3700832 |
| 198 | Neu5Acα2-3Galβ1-3(Neu5Acα2-3Galβ1-4)GlcNAcβ-Sp8 | 128.063375 | 96.74762354 |
| 199 | Fucα1-2Galβ1-4(Fucα1-3)GlcNAcβ–Sp0 | 127.177375 | 24.19515867 |
| 200 | Neu5Acα2-3Galb1-3(Fucα1-4)GlcNAcβ–Sp8 | 126.021025 | 27.23433107 |
| 201 | Neu5Acα2-8Neu5Acα2-8Neu5Acα-Sp8 | 124.4144 | 42.58507407 |
| 202 | Neu5Acα2-3Galβ1-4(Fucα1-3)GlcNAcβ–Sp0 | 124.065025 | 23.80534161 |
| 203 | Galβ1-3(GlcNAcβ1-6)GalNAcα-Sp8 | 122.0201 | 23.08938471 |
| 204 | Neu5Acα2-3Galβ1-4Glcβ–Sp8 | 119.9653 | 47.72912374 |
| 205 | Neu5Acα2-3GalNAcα–Sp8 | 119.7801 | 23.01927533 |
| 206 | Neu5Acα2-3Galβ1-3(6OSO3)GlcNAc-Sp8 | 119.5278 | 26.68268686 |
| 207 | Neu5Acα2-3(GalNAcβ1-4)Galβ1-4Glcβ–Sp0 | 114.442575 | 21.29810975 |
| 208 | GlcNAcβ1-3(GlcNAcβ1-6)Galb1-4GlcNAcb–Sp8 | 113.788725 | 52.74425309 |
| 209 | GalNAca1-3(Fuca1-2)Galb1-4Glcb-Sp0 | 113.584175 | 41.38890104 |
| 210 | Neu5Acα1-2–Sp82 | 112.30095 | 57.39835984 |
| 211 | Glcα1-4Glcβ–Sp8 | 112.261125 | 24.76451871 |
| 212 | Galβ1-4Glcβ–Sp0 | 112.004175 | 29.55100473 |
| 213 | Neu5Gca2-3Galb1-3(Fuca1-4)GlcNAcb-Sp0 | 111.573175 | 52.33524171 |
| 214 | Mana1-6(Mana1-3)Mana1-6(Mana1-3)Manb1-4GlcNAcb1-4 GlcNAcb-Asn | 111.52165 | 44.28755294 |
| 215 | Galβ1-3(Fucα1-4)GlcNAcβ–Sp8 | 110.8326 | 53.52521728 |
| 216 | Galα1-3GalNAcβ–Sp8 | 107.080075 | 61.79718749 |
| 217 | Galβ1-4GlcNAcβ–Sp8 | 106.186 | 33.40593286 |
| 218 | NeuAca2-8NeuAca2-8NeuAca2-8NeuAca2-3(GalNAcb1-4)Galb1-4Glcb-Sp0 | 106.017125 | 15.87690651 |
| 219 | GlcNAcb1-4MDPLys (bacterial cell wall) | 105.39065 | 36.4454086 |
| 220 | Neu5Acα2-6Galβ1-4GlcNAcβ–Sp8 | 105.2932 | 38.95892051 |
| 221 | Galβ1-3(Fucα1-4)GlcNAc–Sp0 | 104.315225 | 62.42564576 |
| 222 | G-ol-amine | 104.26045 | 7.837795784 |
| 223 | Galb1-3GlcNAcb1-3Galb1-4GlcNAcb-Sp0 | 102.78725 | 49.13182413 |
| 224 | Neu5Aca2-3(GalNAcb1-4)Galb1-4GlcNAcb-Sp8 | 102.3985 | 40.40610533 |
| 225 | Neu5Gcα2-3Galβ1-4Glcβ–Sp0 | 101.493825 | 17.92369896 |
| 226 | Fucα1-2Galβ1-4GlcNAcβ–Sp0 | 101.043025 | 18.82605099 |
| 227 | Neu5Gca2-3Galb1-4(Fuca1-3)GlcNAcb-Sp0 | 100.818125 | 12.39795106 |
| 228 | Galβ1-4GlcNAcβ1-6(Galβ1-3)GalNAcα–Sp8 | 100.190225 | 32.02843843 |
| 229 | GlcNAcb1-3Galb1-3GalNAca-Sp8 | 98.801375 | 39.37269169 |
| 230 | Fucα1-2Galβ–Sp8 | 98.49455 | 22.72038425 |
| 231 | GlcNAcβ1-6(Galβ1-3)GalNAcα–Sp8 | 98.0871 | 37.60302921 |
| 232 | Neu5Aca2-8Neu5Aca2-8Neu5Acα2-3Galβ1-4Glcβ–Sp0 | 97.026625 | 29.69958204 |
| 233 | Manα1-2Manα1-2Manα1-3(Manα1-2Manα1-3(Manα1-2Manα1-6)Manα1-6)Manβ1-4GlcNAcβ1-4GlcNAcβ-Asn | 95.6898 | 48.66294402 |
| 234 | Fucα1-4GlcNAcβ–Sp8 | 93.032725 | 24.29174943 |
| 235 | Galβ1-4GlcNAcβ1-3Galβ1-4GlcNAcβ1-3Galβ1-4GlcNAcβ–Sp0 | 92.801375 | 12.06988717 |
| 236 | Galβ1-4GlcNAcβ–Sp0 | 91.687725 | 27.63156793 |
| 237 | Neu5Aca2-3GalNAcb1-4GlcNAcb-Sp0 | 88.237075 | 9.956546181 |
| 238 | Galβ1-4GlcNAcβ1-3GalNAcα–Sp8 | 87.4498 | 34.06045144 |
| 239 | Neu5Acα2-6(Galβ1-3)GalNAcα–Sp8 | 86.756425 | 20.58641314 |
| 240 | Neu5Acα2-3(6-O-Su)Galβ1-4(Fucα1-3)GlcNAcβ–Sp8 | 86.594925 | 28.08988555 |
| 241 | Neu5Aca2-3Galb1-4GlcNAcb1-3Galb1-4GlcNAcb-Sp0 | 85.25035 | 31.83187501 |
| 242 | Mana1-2Mana1-3(Mana1-2Mana1-6)Mana-Sp9 | 82.85235 | 29.66264947 |
| 243 | Neu5Acα2-6Galβ1-4Glcβ–Sp8 | 80.996225 | 33.80971203 |
| 244 | KDNα2-3Galβ1-3GlcNAcβ–Sp0 | 80.666725 | 15.06787614 |
| 245 | Neu5Acα2-3Galβ1-4(Fucα1-3)(6OSO3)GlcNAcβ–Sp8 | 80.287025 | 15.64252668 |
| 246 | Fucα1-2Galβ1-3GlcNAcβ1-3Galβ1-4Glcβ–Sp10 | 79.752075 | 39.17331965 |
| 247 | Galβ1-4GlcNAcβ1-3Galβ1-4Glcβ–Sp0 | 77.8895 | 42.14737956 |
| 248 | (Galβ1-4GlcNAcβ)_2_-3,6-GalNAcα–Sp8 | 76.004325 | 7.999558746 |
| 249 | Neu5Gcα2-3Galβ1-4GlcNAcβ–Sp0 | 72.47185 | 17.20922563 |
| 250 | Glcb1-6Glcb-Sp8 | 67.18885 | 27.92474825 |
| 251 | Galβ1-4(Fucα1-3)GlcNAcb–Sp0 | 63.53735 | 7.09928332 |
| 252 | GlcNAcβ1-6GalNAcα–Sp8 | 62.277625 | 10.8512315 |
| 253 | Neu5Aca2-6Galb1-4GlcNAcb1-3Galb1-4GlcNAcb-Sp0 | 61.06765 | 16.66816398 |
| 254 | Neu5Acα2-6GalNAcα–Sp8 | 57.033425 | 10.4663772 |
| 255 | GlcNAcb1-3Galb1-4GlcNAcb1-3Galb1-4GlcNAcb-Sp0 | 55.3791 | 28.50555364 |
| 256 | Neu5Acα2-8Neu5Acα-Sp8 | 54.899375 | 8.430375858 |
| 257 | GlcNAcβ1-2Galβ1-3GalNAcα–Sp8 | 53.748975 | 11.32629186 |
| 258 | GlcNAcβ1-3GalNAcα–Sp8 | 53.669 | 16.37389153 |
| 259 | Neu5Aca2-3Galb1-4GlcNAcb1-3Galb1-4(Fuca1-3)GlcNAc-Sp0 | 40.7574 | 10.33504556 |
| 260 | Neu5Acα2-6Galβ1-4GlcNAcβ–Sp0 | 37.10405 | 6.109640059 |
| 261 | β-D-Man–Sp8 | 36.5948 | 28.37250746 |
| 262 | GlcNAcb1-3Galb1-4GlcNAcb-Sp8 | 26.95935 | 16.75813078 |
| 263 | Neu5Acα2-3Galβ1-4GlcNAcβ–Sp8 | 26.458225 | 13.24546029 |
| 264 | Neu5Gcα2-6GalNAcα–Sp0 | -0.51165 | 12.61723913 |

**Supplemental materials and methods**

## SAXS

## Protein samples were measured in 25 mM Tris/HCl (pH 7.5) at solute concentrations 1.25, 2.5 and 5.5 mg/ml. The measurements were made at 8°C with 3 min exposure time on a MAR345 image plate detector. The sample–detector distance of 2.7 m covered the range of momentum transfer of 0.12 < *s* < 4.5 nm^−1^ (*s* = 4π sin (θ)/λ, where 2θ is the scattering angle and λ = 0.15 nm is the X-ray wavelength). Two 3 min exposures were made to establish the absence of any radiation damage. The data were normalized to the intensity of the transmitted beam and scattering that was contributed by the buffer was removed. The difference curves were scaled for concentration and extrapolated to infinite dilution. The forward scattering I(0) and the radius of gyration (R_g_) were computed using the Guinier approximation. All subsequent data processing stages were performed with the program package PRIMUS (1). The distance distribution function p(r) and the maximum particle size D_max_ were computed using the program GNOM (2). The molecular mass (MM) of GbpA_fl_ was estimated by comparing the extrapolated forward scattering I(0) with that of a reference solution made of bovine serum albumin (MM = 66 kDa). Low-resolution models of GbpA_fl_ were built by the program DAMMIF (3), which represent the protein as an assembly of dummy residue (391 residues for GbpA_D1-3_, and 462 residues for GbpA_fl_) inside a search volume defined by a sphere of the diameter D_max_. Starting from a random model, DAMMIN employs simulated annealing (SA) to build a protein-like model that fits the experimental data Iexp(s) to minimize the discrepancy:

1.
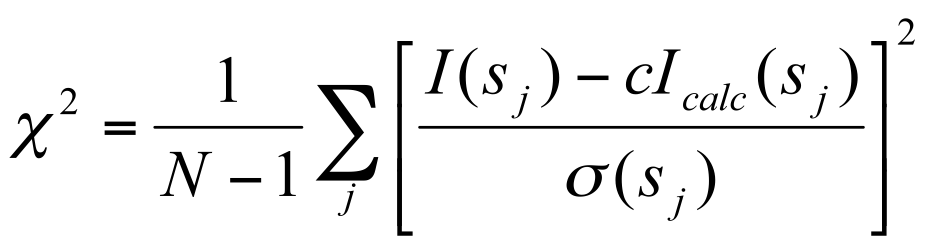

2. where *N* is the number of experimental points, *c* is a scaling factor and *I_calc_(s)* and *σ(s_j_)* are the calculated intensity and the experimental error at the momentum transfer *s_j_*, respectively. An averaged model of GbpA_fl_ was obtained by the superposition of twenty independent DAMMIN reconstructions using the program packages DAMAVER (4, 5) and SUBCOMP (6). A more detailed model of GbpA_fl_ was constructed by rigid body modelling using the program SASREF (7), which employs SA protocol to generate an interconnected assembly of domains without steric clashes fitting the scattering data. The scattering from the individual domains of the crystal structure of GbpAD1-3, and of the homology model of GbpA from amino acids 423 to 485 (GbpAD4) was computed using the program CRYSOL (8). The homology model of GbpAD4 was generated using the program Modeller (9) and the chitin binding module of *Serratia marcescens* chitinase B (PDB ID 1E15; residues 450 – 498) as template (10).

## Circular dichroism

Samples of GbpA (25 mM Tris/HCl pH 7.5) at concentrations of 10 μM (500 μg/ml) were used for CD spectroscopic analysis. All samples were centrifuged at 13000 x g at 4°C for 30 minutes prior to analysis. CD spectra were recorded in a 0.1 cm length quartz cuvette at either 20°C, or the specified temperature, under constant nitrogen flushing using a Jasco J720 spectrophotometer. At least 10 spectra were accumulated, and the appropriate buffer spectra were subtracted. Values were expressed as molar ellipticity, [θ]. The content of β-sheets and α-helices was determined from deconvoluted CD spectroscopic data using the CONTINLL program (11).

**Detection of GbpA in bacterial outer membranes**

Bacterial cells were harvested from 150 ml of culture of each complemented N1RB3 strain (12). The cells were washed twice with 0.1 M HEPES (pH 7) before resuspension in 7.5 ml in the same buffer. Then, each preparation was sonicated (intermittent pulse of 15 seconds for 18 pulses) whilst maintaining temperature at 4 ºC. Intact cells were removed by centrifugation at 7000 x g for 10 minutes. Each supernatant was further centrifuged at 100000 g for 1 hour, and the pellet was resuspended in 0.5% (w/v) N-laurylsarcosine-Na salt for 15 minutes with gentle agitation. As before, each sample was centrifuged at 100000 g for 1 hour to collect the outer membrane protein as pellet. The pellets were resuspended in 0.1 M HEPES.

For all GbpA constructs studied, equal amounts of the preparations were measured by Bradford assay, and confirmed by SDS-PAGE. The proteins were transferred electrophoretically to nitrocellulose membrane (Bio-Rad, USA) under wet condition, at 100 V for 1 hour in chilled transfer buffer (25 mM Tris-HCl, 200 mM glycine, 0.1% SDS and 20% methanol, pH 8.3). The free sites on nitrocellulose membrane were then blocked with 5% (w/v) defatted milk powder dissolved in Tris-buffered saline-0.1% (v/v) Tween 20 (TBS-T, 20 mM Tris-HCl, 500 mM NaCl, pH 7.5) for 2 hours at room temperature. The membrane was then incubated with mouse polyclonal anti-GbpA antiserum (1:1800, v/v) in TBS-T containing 5% defatted milk, and incubated overnight. The secondary antibody used was goat anti-mouse IgG conjugated with horseradish peroxidase (1:5000, v/v) in TBS-T containing 5% defatted milk. Non-specifically bound proteins were removed by washing the membrane with TBS-T twice between the two incubation steps. Immunoreactive components were visualized by chemiluminiscent assay with Immobilon reagent (Millipore).
